# Unraveling the molecular determinants of a rare human mitochondrial disorder caused by the P144L mutation of FDX2

**DOI:** 10.1002/pro.5197

**Published:** 2024-10-28

**Authors:** Deborah Grifagni, Davide Doni, Bianca Susini, Bruno M. Fonseca, Ricardo O. Louro, Paola Costantini, Simone Ciofi‐Baffoni

**Affiliations:** ^1^ Magnetic Resonance Center CERM University of Florence Florence Italy; ^2^ Department of Chemistry University of Florence Florence Italy; ^3^ Department of Biology University of Padova Padova Italy; ^4^ Instituto de Tecnologia Química e Biológica António Xavier (ITQB‐NOVA) Universidade Nova de Lisboa Oeiras Portugal

**Keywords:** FDX2, iron–sulfur protein, ISC machinery, MEOAL, NMR, P144L, rare disease

## Abstract

Episodic mitochondrial myopathy with or without optic atrophy and reversible leukoencephalopathy (MEOAL) is a rare, orphan autosomal recessive disorder caused by mutations in ferredoxin‐2 (FDX2), which is a [2Fe‐2S] cluster‐binding protein participating in the formation of iron–sulfur clusters in mitochondria. In this biosynthetic pathway, FDX2 works as electron donor to promote the assembly of both [2Fe‐2S] and [4Fe‐4S] clusters. A recently identified missense mutation of MEOAL is the homozygous mutation c.431C>T (p.P144L) described in six patients from two unrelated families. This mutation alters a highly conserved proline residue located in a loop of FDX2 that is distant from the [2Fe‐2S] cluster. How this Pro to Leu substitution damages iron–sulfur cluster biosynthesis is unknown. In this work, we have first compared the structural, dynamic, cluster binding and redox properties of WT and P144L [2Fe‐2S] FDX2 to have clues on how the pathogenic P144L mutation can perturb the FDX2 function. Then, we have investigated the interaction of both WT and P144L [2Fe‐2S] FDX2 with its physiological electron donor, ferredoxin reductase FDXR, comparing their electron transfer efficiency and protein–protein recognition patterns. Overall, the data indicate that the pathogenic P144L mutation negatively affects the FDXR‐dependent electron transfer pathway from NADPH to FDX2, thereby reducing the capacity of FDX2 in assembling both [2Fe‐2S] and [4Fe‐4S] clusters. Our study also provided solid molecular evidences on the functional role of the C‐terminal tail of FDX2 in the electron transfer between FDX2 and FDXR.

## 1 INTRODUCTION

Electron transfer pathways are fundamental for assembling iron–sulfur (FeS) clusters in the cell (Braymer et al. 2021). In mitochondria, the electron transfer chain composed by ferredoxin‐2 (FDX2), ferredoxin reductase (FDXR), and NADPH is in charge of pumping the electrons required to form both [2Fe‐2S] and [4Fe‐4S] clusters in the iron–sulfur cluster (ISC) assembly machinery (Cai et al. 2017; Lange et al. 2000; Schulz et al. 2023a; Shi et al. 2012). The importance of this electron transfer chain is reflected by severe genetic diseases including mitochondrial myopathy and sensory neuropathies, generated by pathogenic mutations in either human FDX2 or FDXR genes (Camponeschi et al. 2022; Gurgel‐Giannetti et al. 2018; Lebigot et al. 2021; Paul et al. 2017; Spiegel et al. 2014). This work unravels the molecular basis of a pathogenic mutation of FDX2, contributing to provide hints for the development of a therapeutic strategy for MEOAL disease.

FDX2 genetic mutations have been associated to the development of a rare mitochondrial disease, named episodic mitochondrial myopathy with or without optic atrophy and reversible leukoencephalopathy (MEOAL, OMIM number: 251900) (Aggarwal et al. 2022; Gkiourtzis et al. 2023; Gurgel‐Giannetti et al. 2018; Lebigot et al. 2017; Montealegre et al. 2022; Spiegel et al. 2014; Wongkittichote et al. 2024). MEOAL is an ultra‐rare inherited neuromuscular disorder, clinically characterized by childhood onset of progressive muscle weakness and exercise intolerance. Further, more variable features of this disorder may include optic atrophy, reversible or partially reversible leukoencephalopathy, and later onset of a sensory‐motor polyneuropathy. The first case of MEOAL was described in 2014 in a 15‐year‐old patient due to a homozygous mutation affecting the start codon of FDX2 (c.1A>T, p.Met1Leu) and resulting in a severe reduction of the FDX2 protein levels (Spiegel et al. 2014). The same homozygous mutation was found in four patients with a similar muscular phenotype (Aggarwal et al. 2022; Gkiourtzis et al. 2023; Lebigot et al. 2017; Montealegre et al. 2022; Wongkittichote et al. 2024). In these patients, the enzymatic activity of the FeS‐cluster dependent complexes I, II, and III, as well as mitochondrial aconitase, was severely impaired. These findings are consistent with the specific function of FDX2 in the biogenesis of mitochondrial FeS proteins (Schulz et al. 2023b). Moreover, the activity of pyruvate dehydrogenase (PDH) complex was decreased. This is in accord to the role of the FeS‐dependent lipoate synthase enzyme in functionally activating E2 subunit of PDH complex. In 2018, a homozygous missense mutation in FDX2 (c.431C>T, p.Pro144Leu) was described in six patients from two unrelated families showing a neurological phenotype involving optic atrophy and nystagmus developed by age 3, followed by myopathy and recurrent episodes of cramps, myalgia, axonal polyneuropathy and muscle weakness in the first or second decade of life (Gurgel‐Giannetti et al. 2018). While no difference in FDX2 mRNA expression between patients and controls was observed using muscle samples of the patients with homozygous c.431C>T mutation, a severe reduction of FDX2 protein levels was observed in the same patients as compared to controls. Mitochondrial iron accumulation was also observed in this case.

P144 is a highly conserved residue, which, in the crystal structure of [2Fe‐2S] FDX2 (PDB ID 2Y5C), is located in a loop capped by a few residues of the C‐terminal tail. The latter was recently shown to be an important structural element for the mitochondrial FDX2 function (Schulz et al. 2023a), but how this functionality is realized is fully undefined. The loop of FDX2 containing P144 follows the redox partner‐interacting helix F (Schulz et al. 2023a), and is more than 10Å away from the [2Fe‐2S] cluster. These structural features do not provide a clear picture on how the P144L mutation on FDX2 might cause its misfunction in MEOAL. This work thus aims to define the molecular determinant of the MEOAL disorder caused by the homozygous P144L mutation by first characterizing the effect of the P144L mutation on the structural–dynamic and cluster‐binding/redox properties of FDX2, and then on the protein–protein interaction and electron transfer properties between FDX2 and FDXR. Our studies also provide the molecular grounds for identifying the role of the C‐terminal tail of FDX2 in the electron transfer between FDX2 and FDXR, which was found to functionally differentiate FDX2 from FDX1 (Schulz et al. 2023a; Seeber 2002).

## 2 RESULTS

### 2.1 The P144L mutation does not affect the [2Fe‐2S] cluster binding, its redox properties, and the folding stability of FDX2

**FIGURE 1:**
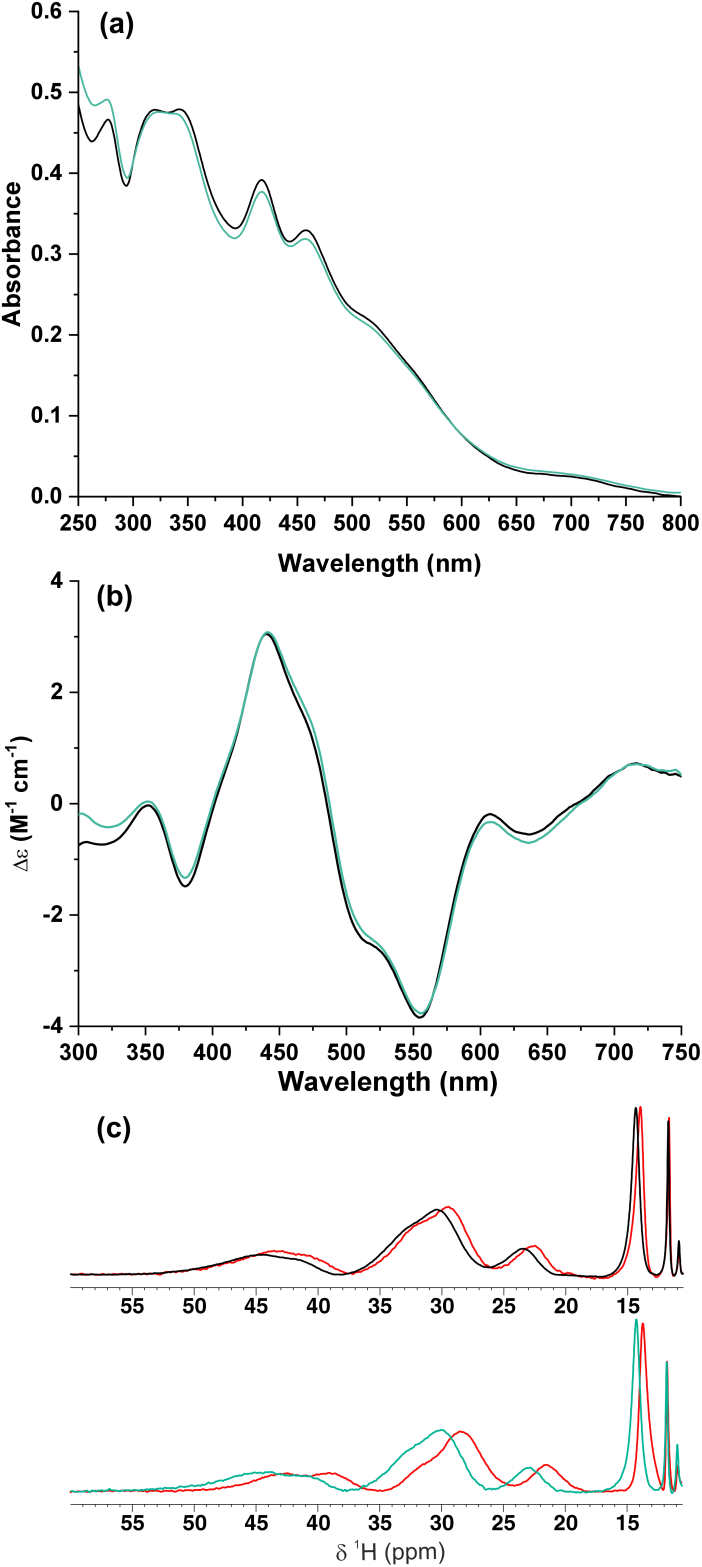
Monitoring the impact of the P144L mutation on the [2Fe‐2S] cluster binding properties. Comparison of UV/visible (a) and UV/visible‐CD (b) spectra of purified WT (black) and P144L (aqua marine) [2Fe‐2S]^2+^ FDX2. Δε values are based on [2Fe‐2S] concentration. (c) 1D ^1^H paramagnetic NMR spectra at 298 K of purified WT (black) and P144L (aqua marine) [2Fe‐2S]^2+^ FDX2, and at 283 K (WT) and 280 K (P144L) in red.

A protein construct of FDX2 comprising residues 69–186 (for both wild‐type and P144L mutant) was expressed in *Escherichia coli* cells. This construct excludes the mitochondrial targeting sequence and the following 13 N‐terminal residues, which have been shown to not having a functional role (Schulz et al. 2023a). The wild‐type (WT) and P144L proteins were anaerobically purified and both proteins were isolated with a bound [2Fe‐2S] cluster at the same level of cluster incorporation (more than 90%, see section 4 for details). This result shows that P144L mutation does not alter the ability of FDX2 to bind the [2Fe‐2S] cluster. To monitor whether the P144L mutation perturbs cluster coordination and its redox properties, UV/visible, UV/visible‐CD, and paramagnetic NMR spectra on both purified proteins were recorded. The UV/visible and UV/visible‐CD spectra of purified WT and P144L [2Fe‐2S] FDX2 are identical and indicative of the presence of a [2Fe‐2S] cluster in an oxidized +2 state (Figure 1a). 1D ^1^H paramagnetic NMR spectra of WT and P144L [2Fe‐2S] FDX2 are also identical and indicative of a [2Fe‐2S]^2+^ cluster bound to Cys ligands (Figure 1b). Both spectra show indeed an anti‐Curie temperature dependence as expected for the presence of two antiferromagnetically coupled Fe^3+^ ions that give rise to a S = 0 ground state of an oxidized [2Fe‐2S]^2+^ cluster (Banci et al. 2018) (Figure 1b). By adding dithionite in excess, the oxidized [2Fe‐2S]^2+^ cluster in both purified WT and P144L FDX2 is fully reduced to generate a [2Fe‐2S]^1+^ cluster as monitored by UV/visible and 1D ^1^H paramagnetic NMR spectra (Figure S1, Supporting Information), which resulted identical for the two proteins. The impact of P144L mutation on the folding stability of [2Fe‐2S]^2+^ FDX2 was finally investigated by NMR performing ^1^H‐^15^N HSQC experiments at increasing temperatures (from 298 to 348 K) on both WT and P144L proteins. From these data, it results that the fold of WT and P144L [2Fe‐2S]^2+^ proteins is destabilized at the same temperature (348 K), as indicated, for both proteins, by the collapse of the amide ^1^H^N^ NMR signal chemical shifts in the range of 7.5–8.5 ppm (Figure S2). This temperature also caused the release of the [2Fe‐2S] cluster from both proteins, as indicated by the lack of the UV/visible bands of the [2Fe‐2S]^2+^ cluster in both samples after treating them at 348 K. Overall, the data clearly indicate that the P144L mutation does not perturb the binding and the coordination environment of the [2Fe‐2S] cluster of FDX2, as well as the cluster redox properties and the folding stability of FDX2.

### 2.2 Both the P144L mutation and the reduction of the [2Fe‐2S] cluster structurally affect helix F and the C‐terminal tail of FDX2

The impact of the P144L mutation on the structural properties of [2Fe‐2S] FDX2 was investigated by solution NMR. ^13^C,^15^N‐labeled WT and P144L [2Fe‐2S] FDX2 samples were prepared and backbone resonance assignments for both proteins in their [2Fe‐2S] cluster‐oxidized and ‐reduced states were performed by running 3D triple resonance experiments. The backbone NMR chemical shifts (δ^15^N, δ^13^C′, δ^13^C^α^, δ^13^C^β^, δ^1^H^α^, and δ^1^H^N^) of both cluster‐oxidized proteins were used to perform secondary structure analysis by TALOS‐N (Shen and Bax 2013), showing that the elements of secondary structure are the same for both proteins and also reproduce those present in the crystal structure of [2Fe‐2S] FDX2. The C‐terminal tail from I172 to H186, which is not present in the crystal structure of [2Fe‐2S] FDX2 since a C‐terminally truncated construct was used to obtain crystals (Schulz et al. 2023a), does not show the presence of secondary structure elements in both WT and P144L proteins. Overall, we can conclude that the P144L mutation does not affect the secondary structure of FDX2.

**FIGURE 2:**
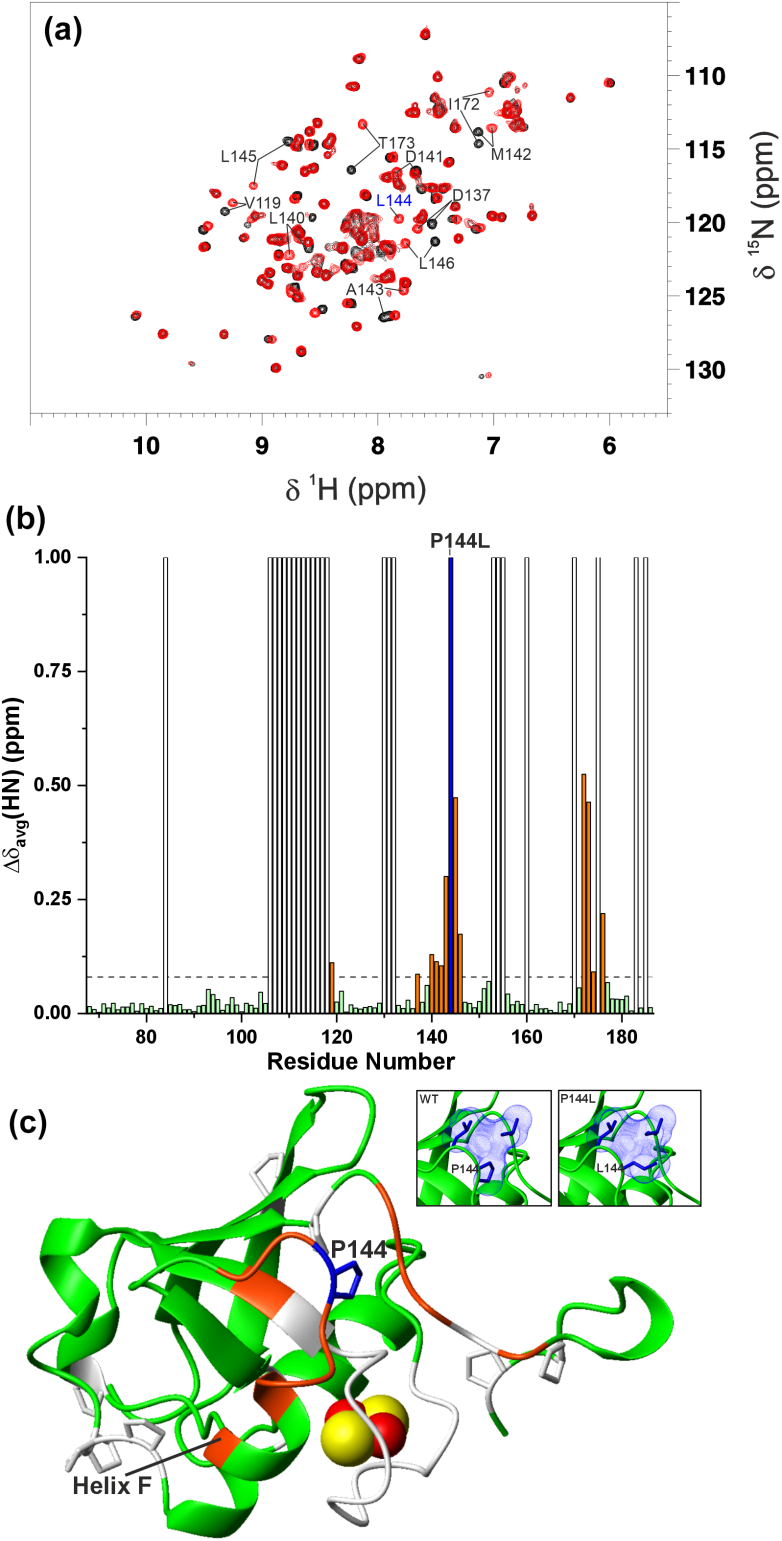
Monitoring the impact of the P144L mutation on [2Fe‐2S]^2+^ FDX2 structure by solution NMR. (a) Overlay of the ^1^H‐^15^N HSQC spectra of WT (black) and P144L (red) [2Fe‐2S]^2+^ FDX2 recorded at 298 K. The signals showing the largest chemical shift differences are indicated. NH signal of L144 is indicated in blue. (b) Backbone weighted average chemical shift differences (Δδ_avg_(HN)) between WT and P144L [2Fe‐2S]^2+^ FDX2. A chemical shift threshold value, indicated as a dashed line, was estimated to define the significant chemical shift differences (see section 4 for details). The white bars indicate proline or unassigned NHs. P144L position is indicated as a blue bar. Orange bars indicate the residues with chemical shift changes larger than the threshold value. (c) The chemical shifts changes larger than the threshold value are mapped in orange on the backbone of the AlphaFold structural model of WT [2Fe‐2S] FDX2 (residues 69–186). The backbone/sidechains of prolines or unassigned residues and of P144 are in white and in blue, respectively. The [2Fe‐2S] cluster is displayed as yellow (sulfur) and red (iron) spheres. Helix F involved in the recognition of redox partners is indicated. In the insets, side‐chain packing involving P144 or L144, L143 and I172 is shown on the structural models of WT and P144L [2Fe‐2S] FDX2, respectively.

The overlay of the ^1^H‐^15^N HSQC spectra of WT and P144L [2Fe‐2S]^2+^ FDX2 showed well‐dispersed backbone NH signals for both proteins, with several NHs showing significantly different chemical shifts between the two proteins (Figure 2a). These data indicate that the P144L mutation, although it does not induce protein unfolding, introduces local structural changes. Weighted‐average backbone chemical shift differences between the two proteins were calculated (Figure 2b) and mapped on the AlphaFold structural model of WT [2Fe‐2S] FDX2 comprising residues 69–186 (Figure 2c), showing that the P144L mutation affects the residues flanking the mutation, which are located in the loop containing P144 and in helix F. The latter is the helix known to be involved in the recognition of redox partners, such as the ferredoxin reductase (Müller et al. 2001). The mutation also affects the chemical shifts of the residues that are spatially close to P144 (Figure 2b,c). In particular, the chemical shifts of the residues from I172 to F176 belonging to the long C‐terminal tail are significantly affected by the mutation, indicating that P144 tightly interacts with this segment of the C‐terminal tail and that the P144L mutation perturbs these interactions. From both the crystal structure and the AlphaFold structural model of WT [2Fe‐2S] FDX2, it results that hydrophobic contacts established between P144 and L143 in the loop and I172 in the C‐terminal tail promote the structural proximity of the first part of the C‐terminal tail to the core domain of FDX2 (Figure 2c, inset). These contacts are also maintained in the AlphaFold structural model of P144L [2Fe‐2S] mutant, since the Pro to Leu residue change is chemically conservative; however, the protein surface is modified by the presence of the longer leucine side‐chain that protrudes to the solvent (Figure 2c, inset).

**FIGURE 3:**
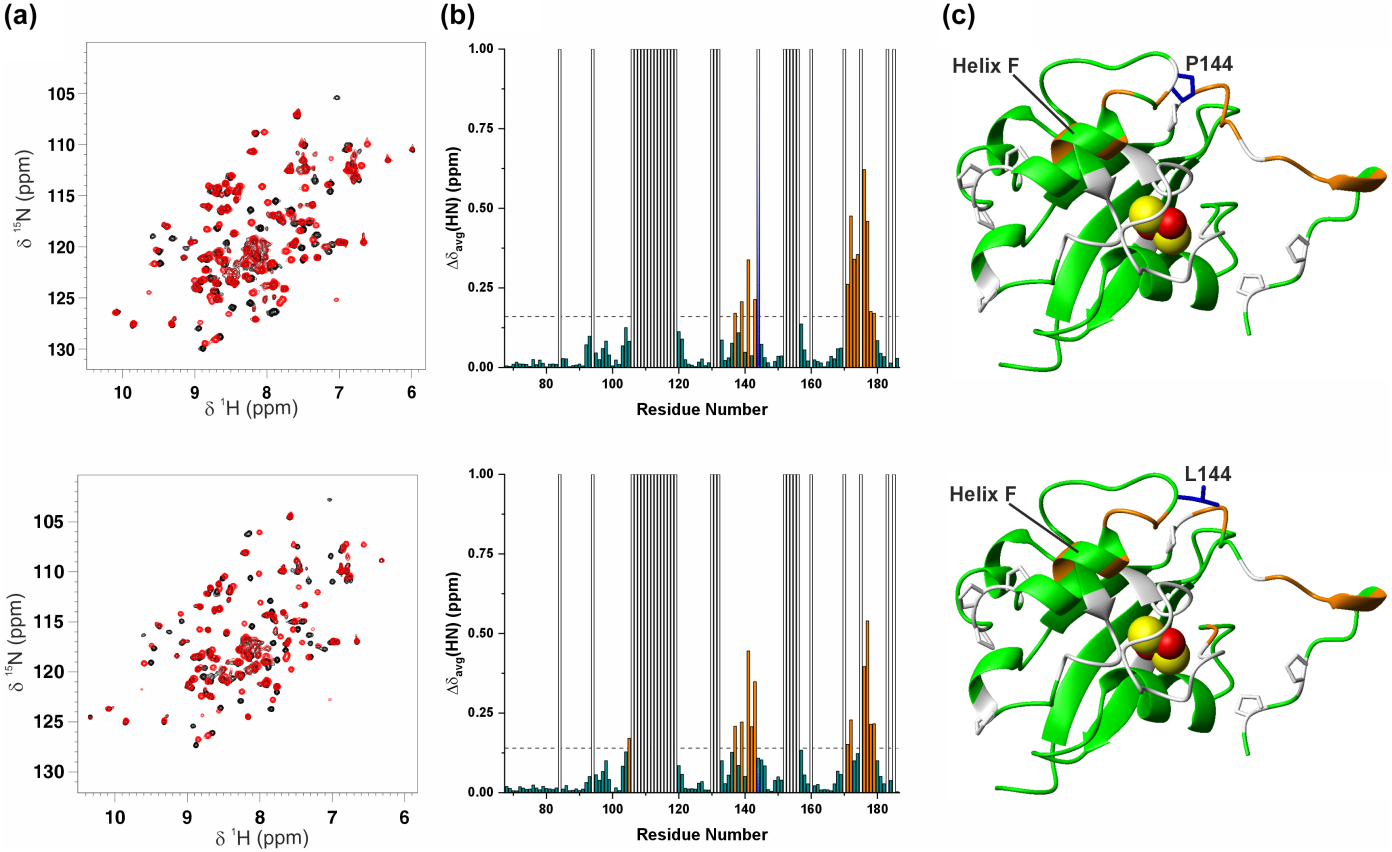
Monitoring redox‐shifts on WT and P144L [2Fe‐2S] FDX2 by solution NMR. (a) Overlay of the ^1^H‐^15^N HSQC spectra of WT (up) and P144L FDX2 (down) with their bound clusters in the oxidized [2Fe‐2S]^2+^ (black) and reduced [2Fe‐2S]^1+^ (red) states, recorded at 298 K. (b) Backbone weighted average chemical shift differences (Δδ_avg_(HN)) between WT (up)/P144L(down) [2Fe‐2S]^2+^ and [2Fe‐2S]^1+^ FDX2. A chemical shift threshold value, indicated as a dashed line in both panels, was estimated to define the significant chemical shift differences (see section 4 for details). The white bars indicate prolines or unassigned NHs. P144 and L144 positions are indicated as shaded blue bars. Orange bars indicate the residues with chemical shift changes larger than the threshold values. (c) The chemical shifts changes larger than the threshold values are mapped in orange on the backbone of the AlphaFold structural models of WT (up) and P144L (down) [2Fe‐2S] FDX2 (residues 69–186). The backbone\sidechains of prolines, and of P144/L144 are in white and in blue, respectively. The [2Fe‐2S] cluster is displayed as yellow (sulfur) and red (iron) spheres. Helix F involved in the recognition of redox partners is indicated.

The effect of [2Fe‐2S] cluster reduction on the structural properties of WT and P144L [2Fe‐2S] FDX2 was then monitored by solution NMR. For both proteins, a comparison of the ^1^H‐^15^N HSQC spectra of cluster‐reduced and ‐oxidized FDX2 (Figure 3a) showed that it is possible to specifically monitor cluster reduction since the chemical shifts of several NH signals are significantly affected by the reduction process, displaying a slow exchange regime on the NMR time scale. The chemical shift differences between the [2Fe‐2S] cluster‐oxidized and ‐reduced species for each protein were calculated and mapped on WT and P144L [2Fe‐2S] FDX2 AlphaFold structural models (Figure 3b,c). From these data, it appears that the largest chemical shift differences involve several residues of the C‐terminal tail (from Lys171 to Gly180) in both P144L and WT FDX2, indicating that a large part of the C‐terminal tail is affected by the structural changes induced by cluster reduction at support of a certain structural proximity of the tail to the redox center (Figure 3b,c). Moreover, residues in helix F monitor the [2Fe‐2S] cluster reduction on both proteins as consequence of structural changes induced by cluster reduction (Figure 3c). The effect of the P144L mutation on the chemical shift changes observed upon cluster reduction in WT and P144L proteins is not dramatic, as it is evident in only three residues (M142 and T173, R174) located near the mutation in the helix F and C‐terminal tail regions, respectively (compare up and down panels of Figure 3b).

### 2.3 The P144L mutation affects the backbone dynamics of helix F and the C‐terminal tail of [2Fe‐2S]^2+^ FDX2

To characterize the impact of the P144L mutation on the backbone dynamics of FDX2, we measured ^15^N relaxation data of WT and P144L [2Fe‐2S]^2+^ FDX2, that is, ^15^N longitudinal (R_1_) and transverse (R_2_) relaxation rates and {^1^H}^15^N heteronuclear NOE values (Figure S3), at a protein concentration of 800 μM. These data were used to calculate, from the R_2_/R_1_ ratio, the overall correlation time for molecular tumbling (τ_m_) and to obtain insights into the motion of the backbone NHs at different time scales by calculating the reduced spectral density function, J(ω), at three frequencies (zero, ωN, and 0.87ωH) (Dayie et al. 1996; Farrow et al. 1995a; O'Sullivan et al. 2009; Peng and Wagner 1995).

The calculated *τ*_m_ values are 7.5 ± 0.9 ns and 7.8 ± 0.4 ns for WT and P144L [2Fe‐2S]^2+^ FDX2, respectively, as expected for a protein of this size in a monomeric state. To investigate whether self‐association occurs in FDX2 as it was found for FDX1 (Behlke et al. 2007; Beilke et al. 2002; Hara and Kimura 1989; Jay et al. 2023; Pikuleva et al. 2000), we performed analytical size exclusion chromatography on both WT and P144L [2Fe‐2S] FDX2 proteins in cluster oxidized and reduced states, varying protein concentration from 80 to 800 μM. Each chromatographic profile showed just a relatively broad peak with its maximum being essentially unperturbed upon dilution with a drift of less than 0.1 mL (Figure S4). These data indicate that [2Fe‐2S] FDX2 is monomeric in all tested conditions and that [2Fe‐2S] FDX2 differs from [2Fe‐2S] FDX1, which, on the contrary, displays a high tendency to self‐associate at high protein concentrations (Behlke et al. 2007). These data also indicate that P144L mutation does not affect the protein assembly.

**FIGURE 4:**
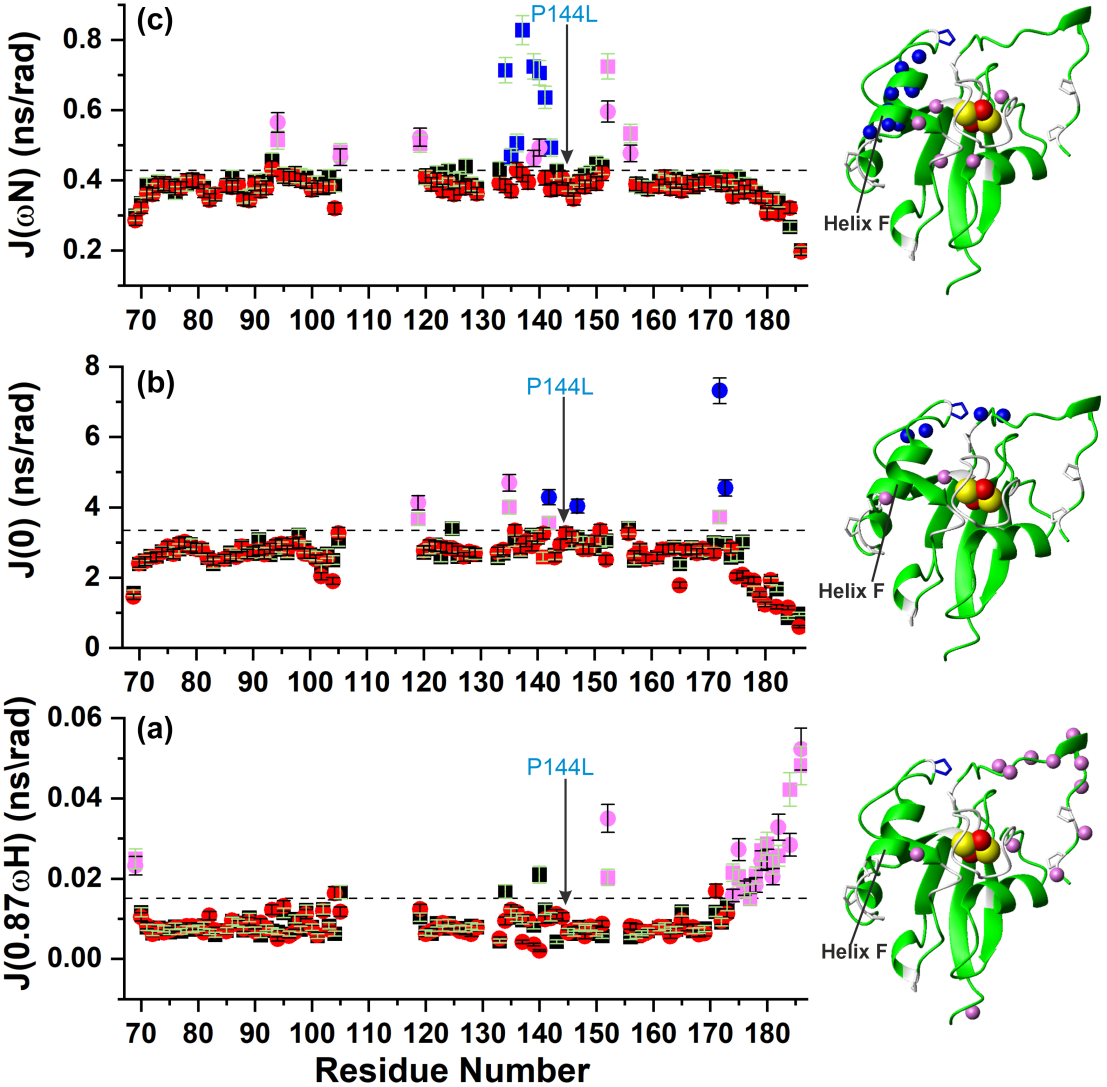
Monitoring the impact of the P144L mutation on the backbone dynamics of [2Fe‐2S]^2+^ FDX2. Reduced spectral density functions J(0.87ωH) (a), J(0) (b), J(ωN) (c) versus the residue number of WT (■) and P144L (●) [2Fe‐2S]^2+^ FDX2, as obtained from the ^15^N relaxation R_1_, R_2_ and {^1^H}^15^N NOE data measured at 500 MHz and 298 K, and at a protein concentration of 800 μM. Threshold values, indicated as dashed lines and obtained by calculating the mean J(ω) values plus 1σ for J(0.87ωH) and J(ωN), and 2σ for J(0), were used to define the significant J(ω) differences. Symbols in violet in (a) indicate the residues with J(0.87ωH) values larger than the threshold values in both proteins. Symbols in violet and blue in (b) and (c) indicate residues with J(0) and J(ωN) values larger than the threshold values in WT or P144L mutant. In (B), the symbols in blue also concerns the residues displaying R_ex_ in P144L [2Fe‐2S]^2+^ FDX2, but not in the WT protein. Backbone NHs having square/circle symbols in violet and blue in the J(ω) panels are mapped as violet and blue spheres on the AlphaFold structural model of WT [2Fe‐2S] FDX2 (residues 69–186). The backbone\sidechains of prolines, and of P144 are in white and in blue, respectively. The [2Fe‐2S] cluster is displayed as yellow (sulfur) and red (iron) spheres. Helix F involved in the recognition of redox partners is indicated.

The high‐frequency spectral densities J(0.87ωH) are particularly useful in indicating, when they increase, fast pico‐second motions typically observed in high flexible regions of proteins (O'Sullivan et al. 2009). It is clear that both WT and P144L [2Fe‐2S]^2+^ FDX2 proteins display meaningfully increased J(0.87ωH) values localized to the C‐terminus (for residues 174–186, shown in violet of Figure 4a), indicating fast backbone fluxionality for this segment typical of intrinsically disordered regions. The high flexibility of the C‐terminal tail is confirmed by the low {^1^H}^15^N heteronuclear NOE values of the residues from R174 to H186 on both proteins (Figure S3). On the contrary, the 70–173 segment shows no extensive fast time‐scale motions, with just two residues showing meaningfully increased J(0.87ωH) on both proteins (shown in violet of Figure 4a). Moreover, comparing J(0.87ωH) values of WT and P144L [2Fe‐2S]^2+^ FDX2 (Figure 4a), it results clear that they have a very similar trend, with just five residues having J(0.87ωH) values larger than the J(0.87ωH) mean plus one standard deviation in WT or P144L [2Fe‐2S]^2+^ FDX2, indicating that the P144L mutation does not essentially affect backbone motions on a fast time scale. A value of the spectral density function J(0) less than two‐fifths of the τ_m_ value indicates internal flexibility of the backbone NHs in sub‐nanosecond time scale (O'Sullivan et al. 2009). This decrease considerably occurs for the N‐terminal D69 and the C‐terminal tail (residues 175–186) on both proteins (Figure 4b), mirroring the J(0.87ωH) spectral densities of these residues (Figure 4a). In addition to sub‐nanosecond flexibility of NHs, slow microsecond to millisecond motions can be also reflected in J(0) spectral densities, as an increase in J(0) values (O'Sullivan et al. 2009). While, for WT [2Fe‐2S]^2+^ FDX2, four residues can be identified as having J(0) values greater than the bulk of the protein (shown in violet squares of Figure 4b), six residues of P144L [2Fe‐2S]^2+^ FDX2 display greater J(0) values (shown in violet and blue circles of Figure 4b). The data are consistent with ^15^N‐R_2_ values for these residues sizably greater than the ^15^N‐R_2_ mean calculated on both WT and P144L [2Fe‐2S]^2+^ FDX2 (Figure S3), indicating a contribution to the measured ^15^N‐R_2_ value caused by the presence of conformational exchange on the millisecond to microsecond time scale. Furthermore, all the six residues of P144L [2Fe‐2S]^2+^ FDX2 display J(0) values significantly greater than the corresponding ones of WT [2Fe‐2S]^2+^ FDX2, indicating a higher conformational backbone fluxionality of these residues induced by the mutation. Four out of these six residues are located close to L144 (Figure 4b), including I172 and T173 at the C‐terminal tail, which are in tight contact with L144 and undergo the largest increases of slow conformational motions on the μs–ms timescale upon the introduction the P144L mutation (shown in blue circles of Figure 4b). These data support the model that the P144L mutation promotes substantial segmental rearrangement and dynamic motions of the portion of the C‐terminal tail in contact with the mutation in P144L [2Fe‐2S]^2+^ FDX2. The presence of a R_ex_ contribution was directly evaluated by measuring the transverse relaxation rates, R_2_, of the backbone amide nitrogens as a function of the τ_CPMG_ duration, which produces different effective field strengths, ν_eff_, as already described in the literature (Arnesano et al. 2001). By comparing these data between WT and P144L [2Fe‐2S]^2+^ FDX2, it results that four residues (M142, Q147, I172, and T173), which display greater J(0) values in P144L mutant than in WT and which are all spatially very close to the mutation, show a substantial different dependence of R_2_ on ν_eff_ (shown in blue circles of Figure 4b). They show indeed a decay dependence of R_2_ on ν_eff_ in P144L mutant but not in the WT protein, corroborating that the P144L mutation promotes conformational backbone motions on the μs–ms timescale in its proximity (Figure S5). J(ωN) values of WT and P144L [2Fe‐2S]^2+^ FDX2 are homogenous with the exception of the N‐ and C‐terminal tails, which show a decrease of the J(ωN) values (Figure 4c), and of a number of residues in the 70–173 segment, which, show an increase of their J(ωN) values (shown in violet and blue circles/squares of Figure 4c). While the decrease of the J(ωN) values for the N‐ and C‐termini still monitors the presence of fast pico‐nanosecond motions of these segments, the increase the J(ωN) values is produced by the paramagnetic relaxation enhancement effect on the ^15^N nuclei (Grifagni et al. 2023; Invernici et al. 2020). Indeed, J(ωN) density function is principally affected by the R_1_ value, which is, for these residues in the 70–173 segment, greatly increased with respect to the ^15^N‐R_1_ mean value as a consequence of a dominant dipolar contribution to the longitudinal paramagnetic relaxation rate (Figure S3) (Nadaud et al. 2011). In agreement to this conclusion, once the residues with increased J(ωN) values are mapped on FDX2 protein structure (backbone NHs shown in violet and blue spheres in Figure 4c), they all surround the blind sphere around the paramagnetic [2Fe‐2S]^2+^ center. A very important aspect concerns the residues of helix F that display a very large increase of both J(ωN) and ^15^N‐R_1_ values in WT [2Fe‐2S]^2+^ FDX2 (shown in blue in Figure 4c), which is quenched in P144L [2Fe‐2S]^2+^ FDX2. This result supports the proposal that the P144L mutation modifies the position of helix F with respect to the paramagnetic center in a way that it moves the helix more distant from the [2Fe‐2S] cluster, being thus the ^15^N‐R_1_ rates of the α‐helical residues no longer affected by the proximity to the paramagnetic center.

### 2.4 The P144L mutation promotes the formation of an aberrant complex between [2Fe‐2S]^2+^ FDX2 and FDXR

**FIGURE 5:**
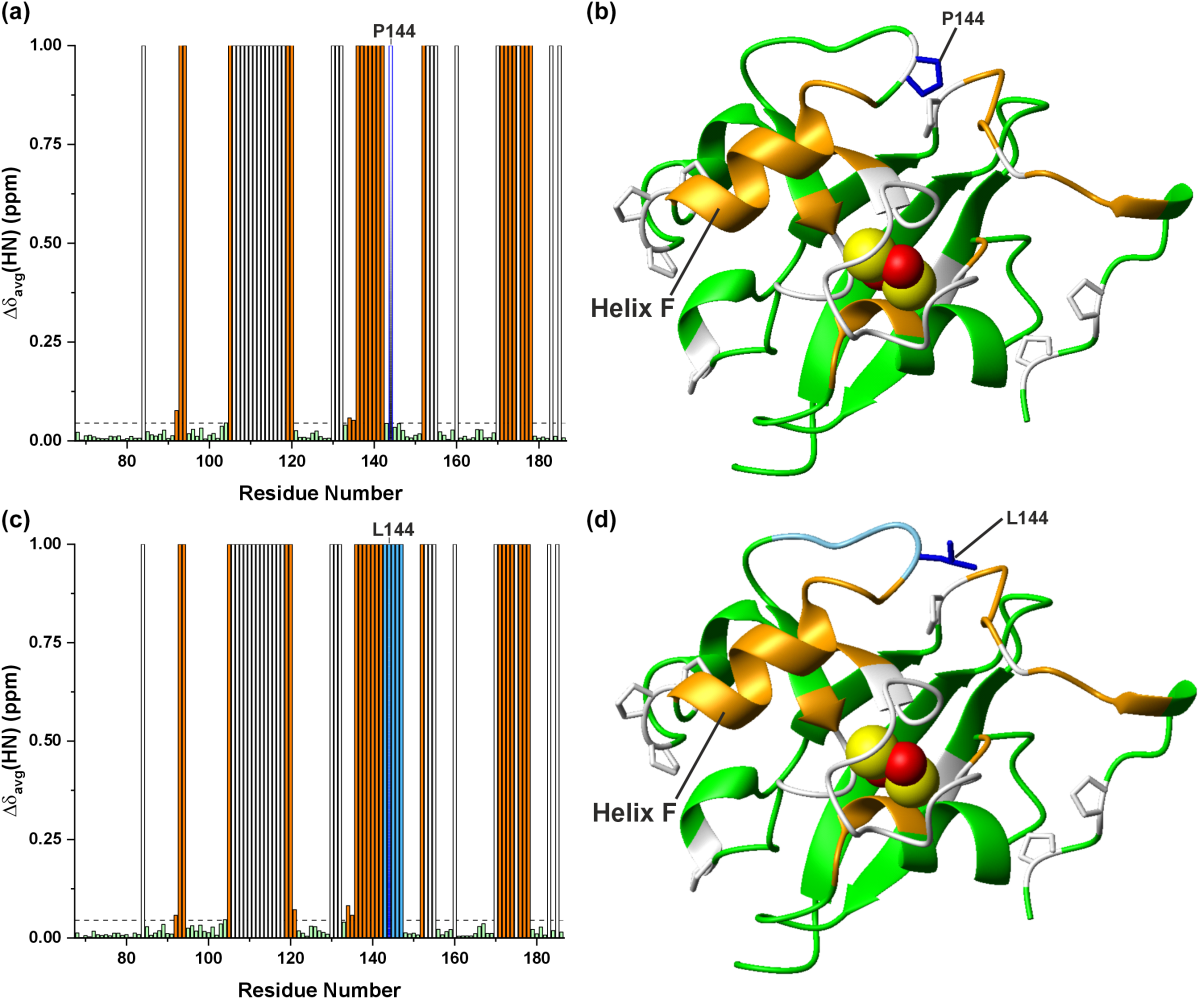
Monitoring the impact of the P144L mutation on the interaction between [2Fe‐2S]^2+^ FDX2 and FAD‐oxidized FDXR. Backbone weighted average chemical shift differences (Δδ_avg_(HN)) between ^15^N‐labeled WT (A) and P144L (C) [2Fe‐2S]^2+^ FDX2 and its 1:1 mixture with unlabeled FDXR_ox_. Orange and sky‐blue bars with Δδ_avg_(HN) = 1 indicate residues whose backbone NH signals broaden beyond detection upon protein–protein interaction. A chemical shift threshold value, indicated as a dashed line in both panels, was estimated to define the significant chemical shift differences (see section 4 for details), where orange bars indicate the residues with chemical shift changes larger than the threshold values. The white bars indicate prolines or unassigned NHs. The chemical shift changes displayed with orange and sky‐blue bars in panels (a) and (c) are mapped with the same colors on the backbone of the AlphaFold structural models of WT (b) and P144L (d) [2Fe‐2S] FDX2 (residues 69–186). The backbone\sidechains of prolines, and of P144/L144 are in white and in blue, respectively. The [2Fe‐2S] cluster is displayed as yellow (sulfur) and red (iron) spheres. Helix F involved in the recognition of redox partners is indicated.

In order to investigate whether the structural‐dynamic changes introduced by the P144L mutation affect FDX2 function, we have characterized, by solution NMR and ITC, the interaction of both WT and P144L FDX2 with the FDX2 redox partner, FDXR. A titration between ^15^N‐labeled [2Fe‐2S]^2+^ FDX2 (WT or P144L) with unlabeled FAD‐oxidized FDXR (FDXR_ox_, hereafter) was performed and the protein–protein interaction was monitored by recording ^1^H‐^15^N HSQC NMR experiments. Upon stepwise additions of FDXR_ox_ to ^15^N‐labeled [2Fe‐2S]^2+^ WT FDX2 from 0.3 to 1.2 mol equiv., we observed chemical shift changes on the ^1^H‐^15^N HSQC maps. These chemical shift variations are in the fast/intermediate exchange regimes on the NMR time scale, indicating the formation of a complex with a moderate affinity between the two proteins. The majority of NH signals of WT [2Fe‐2S]^2+^ FDX2 broaden beyond detection along FDXR_ox_ additions (Figure 5a). In addition, small chemical shift perturbations of backbone NHs (Δδ_avg_(HN)) can be observed for a few residues located next to the NHs broadened beyond detection (Figure 5a). All the chemical shift perturbations can be easily followed in the ^1^H‐^15^N HSQC experiments of the titration, allowing us to map the FDX2 interaction surface upon WT [2Fe‐2S]^2+^ FDX2‐FDXR_ox_ complex formation. From the binding map, it is clear that the [2Fe‐2S] cluster binding site region of WT FDX2 binds to FDXR_ox_, as indicated by the circle of chemical shift perturbations surrounding the [2Fe‐2S] cluster (Figure 5b). Remarkably, helix F is involved in the protein–protein recognition and also the C‐terminal tail of WT [2Fe‐2S]^2+^ FDX2 contributes to the interaction with FDXR_ox_ (Figure 5b). The latter finding is in accord with functional data that recently showed that the C‐terminal tail of FDX2 plays a crucial role in its function (Schulz et al. 2023a). To quantitatively estimate the dissociation constant of the WT [2Fe‐2S]^2+^ FDX2‐FDXR_ox_ complex, isothermal titration calorimetry (ITC) measurements were performed. We found a K_d_ value of 1.04 × 10^−5^M (Figure S6), which is higher by a factor of about 10 with respect to K_d_ values estimated, by optical biosensor system (SPR), for bovine [2Fe‐2S]^2+^ FDX1‐FDXR_ox_ complex formation, indicating a weaker affinity of WT [2Fe‐2S]^2+^ FDX2 toward FDXR_ox_ than [2Fe‐2S]^2+^ FDX1 (Ivanov et al. 1999; Schiffler et al. 2004).

A specular titration was performed with ^15^N‐labeled [2Fe‐2S]^2+^ P144L FDX2 and unlabeled FDXR_ox_. The NMR data showed many similarities but also significant differences. Indeed, a fast/intermediate exchange regime on the NMR time scale was observed for the NH signals affected by protein–protein recognition, as it occurs in WT [2Fe‐2S]^2+^ FDX2‐FDXR_ox_ complex formation. However, once these chemical shift variations are mapped on the protein structure of [2Fe‐2S]^2+^ FDX2, it results that, although [2Fe‐2S]^2+^ P144L FDX2 still binds with its [2Fe‐2S] cluster binding site region toward FDXR_ox_ involving interacting residues similar to those of WT [2Fe‐2S]^2+^ FDX2, the mutated L144 and its surrounding residues also contribute to the interaction surface with FDXR_ox_ (Figure 5a,b vs. Figure 5c,d). This is different from what occurs in WT [2Fe‐2S]^2+^ FDX2‐FDXR_ox_ complex formation, whose P144 and surrounding residues are, indeed, not affected by chemical shift changes and thus not involved in the protein–protein recognition (Figure 5a,b vs. Figure 5c,d). ITC data were then acquired with [2Fe‐2S]^2+^ P144L FDX2 and FDXR_ox_ to monitor the impact of P144L mutation on the [2Fe‐2S]^2+^ FDX2‐FDXR_ox_ complex formation. We found a K_d_ value of 6.66 × 10^−7^M (Figure S6), which is lower by a factor of 15 with respect to the K_d_ value of WT [2Fe‐2S]^2+^ FDX2‐FDXR_ox_ complex, indicating that the P144L mutation stabilizes complex formation. Using the measured K_d_ values of WT and P144L [2Fe‐2S]^2+^ FDX2 and considering that the k_on_ value is between 10^5^ and 10^8^ M^−1^ s^−1^ (this range was reported for transient protein–protein interactions) (Schreiber et al. 2009), it is possible to estimate the k_off_ value, whose reciprocal provide us the residence time of the protein in the complex. Considering a k_on_ value of 10^8^–10^6^ M^−1^ s^−1^, we can estimate that the residence times for WT [2Fe‐2S]^2+^ FDX2 and for P144L [2Fe‐2S]^2+^ FDX2 range between 0.96 and 96 ms and between 15 ms and 1.5 s, respectively. These values clearly fall in the range of transient interactions, that was estimated to be between 10 s and 0.1 ms (De Keersmaecker et al. 2018). In conclusion, all the data showed that the P144L mutation modifies molecular recognition between [2Fe‐2S]^2+^ FDX2 and FDXR_ox_ involving L144 region in the protein–protein recognition, and that this wider protein–protein interaction surface stabilizes the interaction between the two proteins. Thus, the P144L mutation promotes the formation of an aberrant complex between [2Fe‐2S]^2+^ FDX2 and FDXR.

### 2.5 The P144L mutation disrupts the NADPH‐FDXR‐FDX2 electron transfer pathway

**FIGURE 6:**
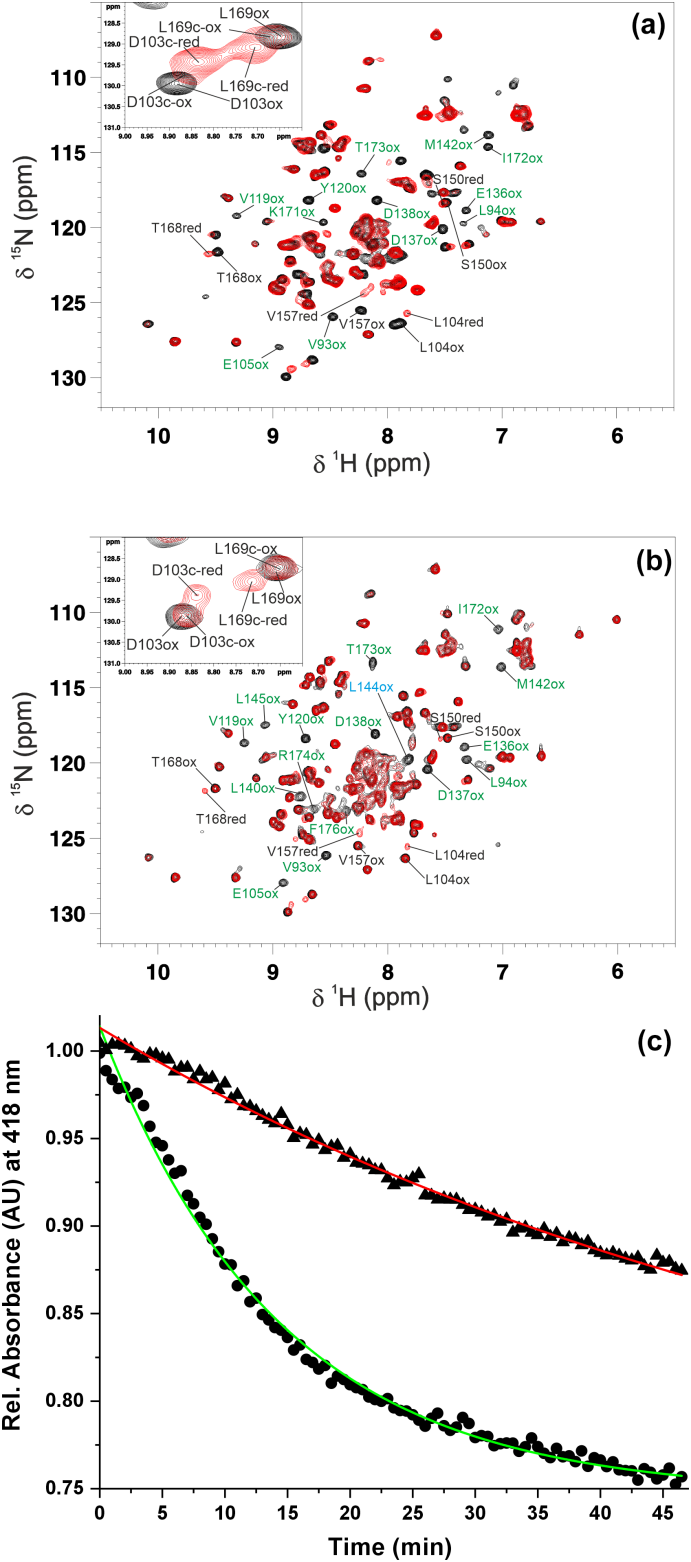
Monitoring the impact of the P144L mutation on the electron transfer between NADPH‐reduced FDXR and [2Fe‐2S]^2+^ FDX2. Overlay of the ^1^H‐^15^N HSQC spectra of WT (a) and P144L (b) [2Fe‐2S]^2+^ FDX2 before (black) and after (red) the addition of 1 eq. of FDXR_ox_ and an excess of NADPH. The NH signals of the residues of oxidized (ox) [2Fe‐2S]^2+^ FDX2 monitoring complex formation by their broaden beyond detection effects are indicated in green. The NH signals of the residues monitoring [2Fe‐2S] cluster reduction in the complex are indicated in black. The latter signals are in a slow exchange on the NMR time scale and both cluster‐reduced and ‐oxidized complexed species (c‐red and c‐ox, respectively) are indicated for each residue. In the inset, two of these residues monitoring both complex formation and [2Fe‐2S] cluster reduction effects are highlighted. (c) Cluster‐reduction of WT (•) and P144L (▲) [2Fe‐2S]^2+^ FDX2 (25 μM) by adding catalytic amounts of FDXR_ox_ (3 μM) and an excess of NADPH (300 μM). The reaction was followed at 418 nm, the absorbance maximum of oxidized [2Fe‐2S]^2+^ FDX2. The mono‐exponential fits are given as red and green lines for WT and P144L [2Fe‐2S]^2+^FDX2, respectively; the correlation coefficients are R‐square = 0.99.

To address whether the aberrant complex formation between P144L [2Fe‐2S]^2+^ FDX2 and FDXR affects the electron transfer chain that drives the electrons from NADPH to FDX2 via FDXR, we have prepared mixtures containing ^15^N‐labeled [2Fe‐2S]^2+^ FDX2 (WT or P144L) and FDXR at 1:0.5 and 1:1 ratio, with an excess of NADPH. The ^1^H‐^15^N HSQC spectra of these mixtures were recorded and compared with those of the isolated ^15^N‐labeled [2Fe‐2S]^2+^ FDX2 (WT or P144L) proteins, respectively (Figure 6a,b).

NMR data analysis of [2Fe‐2S]^2+^ WT FDX2‐FDXR‐NADPH 1:1 mixture showed that FDX2 is not free in solution but is fully complexed with FDXR and displays molecular recognition with FDXR as that observed in the oxidized [2Fe‐2S]^2+^ WT FDX2‐FDXR_ox_ complex formation. Indeed, the backbone NHs broadened beyond detection upon the protein–protein interaction between oxidized [2Fe‐2S]^2+^ WT FDX2 and FDXR_ox_ undergo the same broadening effects in the [2Fe‐2S]^2+^ WT FDX2‐FDXR‐NADPH mixture (see some of them marked in green in Figure 6a). In the case that uncomplexed, reduced [2Fe‐2S]^+^ WT FDX2 was formed in the mixture, these signals should have been observed because they are detected in the isolated cluster‐reduced FDX2 protein; however, they were not. In addition, some NH signals allow us to monitor the reduction of the [2Fe‐2S] cluster of FDX2. Indeed, in the ^1^H‐^15^N HSQC spectrum of the [2Fe‐2S]^2+^ WT FDX2‐FDXR‐NADPH mixture, there are also NH signals whose chemical shifts are perturbed in a slow exchange regime on the NMR time scale due to the change of the redox state of the [2Fe‐2S] cluster and are, in contrast, little affected by protein–protein interaction (see some of them marked in black in Figure 6a and in the inset). From this set of signals, it results that the [2Fe‐2S] cluster of FDX2 complexed with FDXR is 90% reduced. In the inset of Figure 6a, we can indeed observe, for each residue, an intense signal corresponding to a major form of the FDX2‐FDXR complex with chemical shifts typical of cluster‐reduced FDX2 (named L169c‐red and D103c‐red in the inset of Figure 6a) and a very weak signal corresponding to a minor form with chemical shifts typical of [2Fe‐2S]^2+^ WT FDX2 complexed with FDXR_ox_ (named L169c‐ox and D103c‐ox in the inset of Figure 6a). Lastly, the NMR data showed that the presence of NADPH in the mixture does not significantly modify how [2Fe‐2S] FDX2 interacts with FDXR. Indeed, the chemical shift variations observed in the [2Fe‐2S]^2+^ WT FDX2‐FDXR‐NADPH mixture are essentially a combination of two effects only, that is, [2Fe‐2S] cluster reduction of FDX2 and complex formation between [2Fe‐2S] FDX2 and FDXR. This result is in agreement with the structural data on FDXR, which showed that NADPH/NADP^+^ binding essentially does not promote conformational changes of the NADP and FAD domains of FDXR, and thus FDXR‐FAD domain recognizes [2Fe‐2S] WT FDX2 in the same way independently by the presence of NADPH/NADP^+^ cofactors (Ziegler and Schulz 2000). In conclusion, the NMR data showed that, once NADPH is available in solution, [2Fe‐2S] FDX2 is largely reduced by FDXR thanks to the formation of a complex where a specific protein–protein interface drives electron transfer.

NMR data analysis of [2Fe‐2S]^2+^ P144L FDX2‐FDXR‐NADPH 1:1 mixture showed complex formation still occurring between the two proteins similarly to what was observed in the oxidized [2Fe‐2S]^2+^ P144L FDX2‐FDXR_ox_ complex, where the mutated L144 and its surrounding residues still participate to the interaction surface with FDXR to form the same aberrant complex. The NH signals monitoring protein–protein interaction between oxidized [2Fe‐2S]^2+^ P144L FDX2 and FDXR_ox_ (some of them are marked in green in Figure 6b) are, indeed, perturbed in the same way in the [2Fe‐2S]^2+^ P144L FDX2‐FDXR‐NADPH mixture, showing the same line broadening beyond detection effects. A marked difference is, however, observed in the [2Fe‐2S] cluster reduction of P144L FDX2. Indeed, the NH signals of P144L FDX2 monitoring the redox process indicated a poor reduction of the [2Fe‐2S] cluster. Once quantified by integrating the NH signals that discriminate the cluster‐oxidized (c‐ox in the inset of Figure 6b) and ‐reduced (c‐red in the inset of Figure 6b) forms of complexed FDX2, the [2Fe‐2S] cluster of P144L FDX2 resulted to be 31% ± 5 reduced only. In conclusion, the NMR data of the two mixtures clearly indicated that the aberrant complex formation induced by the P144L mutation generates a decreased yield of cluster reduction of FDX2.

As a final point of the study of the effect of the P144L mutation in the electron transfer chain, the rate of the [2Fe‐2S] cluster reduction was monitored by UV/visible spectroscopy following the change over time in absorbance at 418 nm for both [2Fe‐2S]^2+^ FDX2 (WT or P144L)‐FDXR‐NADPH mixtures. The absorption band at 418 nm decreases its intensity in both cases but the trend of decrease is different between WT and P144L FDX2 mixtures. The reduction rate of the [2Fe‐2S] cluster in P144L FDX2 is, indeed, 4.5 times slower than that of WT FDX2 (Figure 6c), indicating that the P144L mutation negatively affects, not only the efficiency of the cluster reduction, but also the kinetics of the reduction process.

## 3 DISCUSSION

Humans like most mammalian species possess two mitochondrial [2Fe‐2S] FDXs, FDX1 (also named adrenodoxin) and FDX2. Although their identification dates back to more than 20 years ago (Seeber 2002), FDX1 and FDX2 have recently attracted considerable attention for two main reasons that concern the involvement of FDX2 in a rare disease (MEOAL) and the molecular specificity of FDXs in their mitochondrial function. Here, we have investigated the first aspect characterizing the P144L pathogenic mutation of FDX2 with the final goal of identifying how the mutation disrupts the mitochondrial function of FDX2.

We showed that P144L mutation does not abolish [2Fe‐2S] cluster binding or even affects the [2Fe‐2S] cluster coordination as well as it does not perturb its redox properties. This agrees with the long distance of P144 from the Cys cluster ligands (more than 10Å). The P144L mutation, however, has an impact on the structure and on the backbone dynamic motions of FDX2, which, although not dramatic, involves regions of the protein important for the function of FDX2. Specifically, the NMR data showed that the P144L mutation modifies the interactions that P144 and its surrounding residues establish with a part of the C‐terminal tail, and that it promotes a backbone conformational fluxionality in the μs–ms timescale for the C‐terminal residues in contact with P144 as well as for some residues surrounding P144. According to R_1_ NMR relaxation data, these P144L‐induced structural–dynamic rearrangements perturb the position of helix F. P144 is indeed located just before helix F, and the R_1_ NMR relaxation data support that, although P144L mutation does not modify the length of helix F, it displaces helix F farther away from the [2Fe‐2S] cluster. Interestingly, NMR data also showed that the [2Fe‐2S] cluster reduction in both WT and P144L proteins alters the structural environment of helix F and the C‐terminal tail. In conclusion, we can state that the P144L mutation impacts at structural and dynamic levels on both helix F and C‐terminal tail, which are the same regions structurally altered by the [2Fe‐2S] cluster reduction in both WT and P144L FDX2 proteins. These findings suggest that the P144L mutation might have a negative impact in the electron transfer function of FDX2 with its protein partners, and our NMR interaction data clearly demonstrate that this occurs. Indeed, the P144L mutant modifies the interaction pattern observed between WT [2Fe‐2S] FDX2 and FDXR by involving in the interaction surface with FDXR also the mutated L144 and its surrounding residues, which do not participate in the interaction with FDXR in the case of the WT protein. This aberrant interaction pattern induced by the mutation has two main effects, to stabilize the complex formation and, most importantly, to decrease the efficiency and the rate of cluster reduction of FDX2 by FDXR. We can also suggest that the conformational backbone motions, which arise in the proximity of L144 due to the P144L mutation (Figure 4b), might drive partner recognition between the P144L mutant and FDXR, thereby promoting the stabilization effect observed for the complex formation of the mutant compared to that of the wild type. The defects of FDX2 reduction by FDXR described here in vitro when Pro 144 is mutated to Leu can elucidate the onset of the MEOAL disease for the patients affected by P144L pathogenic mutation. It has been suggested that the phenotypes of the six patients affected by P144L mutation, all in homozygosity, are a consequence of the misfunction of the FeS cluster assembly of respiratory complexes I‐III, and of heme A production for complex IV. The P144L mutation also causes iron deposition in the cells, similarly to what has been reported in ISCU myopathy (Kollberg et al. 2009; Olsson et al. 2008), in agreement with the role of FDX2 in cellular iron homeostasis via its mitochondrial role in the maturation of cytosolic IRP1 to an aconitase. The P144L mutation also generates an almost complete absence of FDX2 protein in the patient tissue. Our findings indicating that the electron transfer from FDXR to FDX2 is damaged by P144L mutation support a model in which P144L FDX2 is not able to efficiently assemble both [2Fe‐2S] and [4Fe‐4S] cluster in the mitochondrial ISC machinery. However, this function is not fully abolished since our studies showed that FDXR is partially able to reduce P144L FDX2 (~30%), and thus this mutation is not expected to dramatically damage the patients. This agrees with the quite long‐time survival of the patients affected by P144L mutation. Moreover, the almost complete absence of P144L FDX2 in MEOAL patients affected by this mutation (Gurgel‐Giannetti et al. 2018) can be rationalized by our studies. Indeed, the inefficient electron transfer between FDXR and FDX2 might generate low amounts of assembled [2Fe‐2S] cluster in mitochondria. Apo FDX2 might be thus largely abundant. It is expected that apo FDX2 is degraded in mitochondria since the apo form of FDX2 is largely unfolded, as shown here by our NMR studies, and because it is common to observe largely decreased protein levels for mitochondrial FeS target proteins once not assembled by the mitochondrial ISC machinery (Balk et al. 2004; Chen et al. 2002; Kispal et al. 1999; Sheftel et al. 2012). As support of this model, we have observed that the expression of human FDX2 in *E. coli* cells grown with low amounts of iron in the culture media do not produce a soluble FDX2 protein in the cytoplasm, at variance of what was observed when iron was abundant in the culture.

FDXR has been recently associated to a rare disease showing similar phenotypes to those of patients' cells with mutations in FDX2. Our studies are significant for one of the six detected FDXR mutations (R242W). This mutant is unable to complement the *arh1*Δ yeast growth defect, at variance what WT FDXR does (Paul et al. 2017). We showed here that FDX2 primarily recognizes FDXR by its negative patch of helix F and it was shown that R242 of FDXR is one of the crucial residues involved in protein recognition with the negative patch of helix F in the FDX1‐FDXR complex. We can thus propose that the FDX2‐FDXR complex formation might be functionally compromised upon R242W mutation of FDXR similarly to what we have observed for P144L FDX2 mutation. Thus, the R242W mutation might negatively affect electron transfer or recognition between the two proteins, thus damaging the formation of mitochondrial FeS clusters, as observed in MEOAL disorder. The similarities of the phenotypes observed for the P144L FDX2 and R242 FDXR mutants support this model.

Our NMR study showed that WT FDX2 interacts with FDXR forming a complex similarly to what observed in FDX1‐FDXR complex formation (Keizers et al. 2010; Ziegler and Schulz 2000). Our NMR data provide the first clue on the role of the C‐terminal tail (from K171 to V178) in the FDX2‐FDXR recognition. Indeed, while the role of helix F in partner's recognition of FDXs is well established by several works, the role of the C‐terminal tail is still being investigated nowadays. A long debate is indeed present in the literature on the role for the C‐terminal tail in FDXs dimerization (Behlke et al. 2007) with the aim of understand whether and how dimeric FDXs species participate in the electron transfer process with their protein partners (Beilke et al. 2002; Jay et al. 2023; Pikuleva et al. 2000). Interestingly, FDX1 dimerization depends on the oxidation state of the [2Fe‐2S] cluster. Indeed, upon its reduction, the monomeric FDX1 species is formed, while, in the oxidized state, FDX1 dimers are mainly present. Recent data supports that FDX1 interacts with its protein partners in the monomeric form and that the dimeric species regulates the availability of monomers of FDX1 for the FDXR reduction process (Jay et al. 2023). At variance of FDX1, we showed here that no dimerization is observed in FDX2 in all conditions tested by us. The absence of a dimerization effect in FDX2 with respect to FDX1 can be rationalized by a very different C‐terminal region (the last 12 residues) of FDX2 versus that of FDX1, which indicates that the C‐terminal tail of FDX2 is not prone to induce homodimerization. On the other hand, the C‐terminal tail of FDX2 has been recently shown to play a crucial role in FeS protein biogenesis. The C‐terminal tail of FDX2 was shown indeed to be an important structural element for FDX2 functionality as well as to play a role in selecting protein partners receiving electrons from cluster‐reduced FDX2 (Schulz et al. 2023a, 2023b). Our NMR data showed now that WT FDX2 forms a complex with FDXR involving helix F in the protein–protein recognition. This interaction is conserved also in the FDXR‐FDX1 complex. These similar interfaces in the two complexes are consistent with the charge properties of helix F of FDX2, that is strongly negatively charged conserving all Asp and Glu residues of FDX1 interacting with FDXR. Our NMR data also indicate the involvement of the C‐terminal tail (from K171 to V178) in the FDX2‐FDXR recognition. This interaction is loose in the FDX1‐FDXR structure where, indeed, the C‐terminal residues of FDX1 adopt flexible conformations (atomic displacement factors up to 80Å^2^ and or no electron density seen beyond Ala110 of FDX1). NMR data also recently showed that no significant chemical shifts are detected at the C‐terminal tail of FDX1 upon interaction with FDXR, and that the removal of the C‐terminal tail of FDX1 increases the apparent affinity toward FDXR, because of the prevention of the formation of the FDX1 dimer, which likely competes with the FDXR‐FDX1 complex. Comparing our data on FDX2 with those on FDX1, the more realistic model is that the C‐terminal tail of FDX2 interacts with the protein core of FDX2 being not fully independent and in such a way modulates the recognition of FDX2 with its protein partner FDXR. On the contrary, FDX1 exploits the C‐terminal tail for homodimerization, and not for partner recognition, through which it regulates availability of the monomeric FDX1 form interacting with protein partners. The structure of [2Fe‐2S] FDX2 with the whole C‐terminal tail would be required to fully validate this model.

## 4 MATERIALS AND METHODS

### 4.1 Protein expression and purification

DNA sequences codifying for WT FDX2 (69–186) and FDX2 mutant where Pro 144 was mutated in Leu, both containing at the N‐terminus 6xHis‐tag and a following TEV‐cleavage site, were cloned between NdeI and XhoI restriction sites of pET29b(+) and used to transform *E. coli* BL21 (DE3) cells. These constructs excludes the mitochondrial targeting sequence and the following 13 N‐terminal residues, which have been shown to not having a functional role (Schulz et al. 2023a). A transformed colony was inoculated in 100 mL of Luria‐Bertani medium and incubated overnight. 20 mL of preculture were transferred into 1 L of M9 minimal medium supplemented with MgSO_4_ 120 mg L^−1^, CaCl_2_ 33 mg L^−1^, biotin 1 mg L^−1^, thiamine 1 mg L^−1^, kanamycin 50 mg L^−1^, ^13^C‐labeled or unlabeled glucose 3 g L^−1^, ^15^N‐labeled or unlabeled ammonium sulfate 1.2 g L^−1^, and 3 mL L^−1^ of a metal mix solution (HCl 5M 8 mL L^−1^, FeCl_2_·4H_2_O 5 g L^−1^, CaCl_2_·2H_2_O 184 mg L^−1^, H_3_BO_3_ 64 mg L^−1^, CoCl_2_·6H_2_O 18 mg L^−1^, CuCl_2_·2H_2_O 4 mg L^−1^, ZnCl_2_ 340 mg L^−1^, Na_2_MoO_4_·2H_2_O 605 mg L^−1^ and MnCl_2_·4H_2_O 40 mg L^−1^). Cells were incubated at 37°C until OD_600_ reached a value between 0.4 and 0.5 when 250 μM Mohr's salt was added. The protein overexpression was induced, after cooling down the temperature at 20°C for 20 min, with 0.5 mM IPTG at OD_600_ of 0.6–0.8 and cells were incubated overnight. Cells pellet was resuspended in lysis buffer (50 mM Tris, 500 mM NaCl, 15 mM imidazole, pH 8, 5 mM DTT), physically disrupted by sonication and centrifuged at 35,000 rpm for 40 min at 4°C. The soluble fraction was collected, immediately transferred in glove box, and purified by using an HisTrap Fast Flow column previously equilibrated in the lysis buffer. Column washing was performed using 150 mL of lysis buffer, subsequently the protein was eluted using an elution buffer with a high concentration of imidazole (50 mM Tris, 500 mM NaCl, 500 mM imidazole, pH 8). The protein was then exchanged in lysis buffer using a PD‐10 column and 6xHis‐TEV protease (1 mg mL^−1^) was added to the solution. After overnight incubation, the sample was loaded to the HisTrap column to remove the 6xHis tag and 6xHis‐TEV protease. The protein buffer was exchanged in 30 mM HEPES, 150 mM NaCl at pH 7.5 and the protein was stocked at −80°C.

The FDXR expression was performed following two different protocols, one reported in literature (Srour et al. 2022) and one described below. *Escherichia coli* Arctic(DE3) competent cells were transformed with pET28a(+) plasmid containing the FDXR gene (33–491) cloned between NdeI and XhoI. 40 mL of preculture and 1 mL of kanamycin were added to 1 L of culture in TB medium, boosted with 4 mL of glycerol, and put at 37°C. Once OD_600_ equal to 0.6 was reached, the FDXR expression was induced with 1 mM of IPTG, and the culture was supplemented with 280 μM of riboflavin. The cells were left overnight at 12°C. Cells pellet was resuspended in 20 mL of lysis buffer (25 mM Tris, 10% glycerol, 150 mM NaCl, pH 8) and lysed by sonication. The soluble and insoluble components were separated by ultracentrifuge at 35,000 rpm for 40 min at 4°C. The soluble fraction was loaded into a 5 mL HisTrap Fast Flow column and was washed with 75 mL of binding buffer. Purification was done via a linear gradient from 0% at 100% imidazole 0–250 mM in 150 mL, and the protein eluted at an imidazole concentration of 100 mM. A PD‐10 column was finally performed to change the buffer in 30 mM HEPES, 150 mM NaCl at pH 7.5.

### 4.2 Analytical methods and absorption electronic spectroscopies

Analytical size exclusion chromatography was performed on purified samples with a Superdex 200 Increase 10/300 GL column attached to an AKTA pure chromatography unit using a continuous flow rate of 0.5 mL min^−1^. The column was calibrated with gel filtration marker calibration kit, 6500–66,000 Da (Sigma‐Aldrich), to obtain the apparent molecular masses of the detected species. The column was equilibrated with nitrogen‐purged 30 mM HEPES, 150 mM NaCl at pH 7.5.

UV/visible absorption (250–800 nm) and UV/visible‐CD (300–750 nm) spectra were recorded at 298 K using a Cary 50 Eclipse spectrophotometer and a JASCO J‐810 spectropolarimeter, respectively. The purified WT and P144L protein samples were in 50 mM phosphate buffer, 150 mM NaCl at pH 7.0 or in 30 mM HEPES buffer, 150 mM NaCl pH 7.5 at a concentration of 100 μM in a 1 cm path length sealed cuvette. All spectroscopy data shown are representative of three or more independent experiments.

### 4.3 NMR spectroscopy

The folding stability of WT and P144L [2Fe‐2S]^2+^ FDX2 proteins at a concentration of 200 μM was evaluated by acquiring a set of 2D ^1^H‐^15^N HSQC spectra at a 700 MHz Bruker spectrometer equipped with a TXI probe at temperatures ranging from 298 to 348 K with a temperature increase of 5 K for each spectrum. NMR buffer is 30 mM HEPES, 150 mM NaCl pH 7.5. UV/visible spectra of the NMR samples treated at 348 K were then collected at room temperature showing no UV/vis signals in a wavelength range of 300–800 nm consistent with cluster release.

1D ^1^H paramagnetic NMR spectra were performed at two temperatures for each cluster‐oxidized sample: 283 and 298 K for WT [2Fe‐2S]^2+^ FDX2 and 280 and 298 K for P144L [2Fe‐2S]^2+^ FDX2. 1D ^1^H paramagnetic NMR spectra were also recorded at 298 K on samples treated with 10 mM dithionite. Spectra were recorded on a Bruker AV400 MHz spectrometer, equipped with a 5‐mm 1H selective high‐power probe without gradients. Experiments were performed with a WEFT pulse sequence (Inubushi and Becker 1983), with acquisition and recycle delays of 34 and 10 ms, respectively, and a dwell time of 3 μs. Squared cosine and exponential multiplications were applied prior to Fourier transformation (Ciofi‐Baffoni et al. 2014). Manual baseline correction was performed, using polynomial functions. Each experiment was successfully repeated at least three times.

Solution NMR experiments for backbone resonance assignment were performed on ^13^C and ^15^N labeled WT and P144L FDX2 in both cluster redox states ([2Fe‐2S]^2+^ and [2Fe‐2S]^+^), in a buffer solution containing 30 mM HEPES, 150 mM NaCl at pH 7.5. HNCA, HN(CO)CA, HN(CA)CO, HNCO, HNCACB, CBCA(CO)NH, HBHA(CBCACO)HN 3D triple resonance experiments and ^15^N‐NOESY‐HSQC experiments were acquired at 700 MHz spectrometer at 298 K. 3D NMR spectra were processed using the standard Bruker software (Topspin 4.4) and analyzed with CARA program (Keller and Wüthrich 2004). Secondary structure analysis was performed by TALOS‐N (Shen and Bax 2015). NMR experiments for measuring the ^15^N relaxation rates were recorded at 298 K on Bruker Avance spectrometers operating at 500 MHz. ^15^N R_1_, ^15^N R_2_, and steady‐state {^1^H}^15^N heteronuclear NOEs were measured with previously described pulse sequences, which employ gradient selection and sensitivity enhancement, as well as minimal water suppression (Farrow et al. 1994). ^15^N R_2_ were measured with a refocusing time (τ_CPMG_) of 450 μs with the Carr‐Purcell‐Meiboom‐Gill (CPMG) sequence (Mulder et al. 1999; Peng and Wagner 1994). In all experiments, the water signal was suppressed with “water flipback” scheme (Grzesiek and Bax 1993). The experimental relaxation rates were used to map the spectral density function values J(0.87ωH), J(ωN), and J(0) (Farrow et al. 1995b) obtained by applying the Bruker Dynamics Center NMR software. R_2_ rates were also measured as a function of the τ_CPMG_ value to monitor the changes of the transverse relaxation rates in the presence of relatively weak effective fields (Kay et al. 1992; Peng and Wagner 1994). Experiments were collected at four CPMG refocusing τ_CPMG_ delays: 450, 600, 850, and 1150 μs. Relaxation delays varied from 7 to 230 ms, the exact values depending on τ_CPMG_. A dependence of R_2_ on the length of τ_CPMG_ delay indicates an exchange contribution R_ex_ to transverse relaxation rate (Orekhov et al. 1994), which has been estimated by an equation in terms of the weak effective magnetic field, ν_eff_, applied in the xy plane and resulting from the CPMG pulse train (see for details, Arnesano et al. (2001)).

NMR titrations were performed to investigate the interaction between WT or P144L [2Fe‐2S] FDX2 and FDXR through acquisition of ^1^H‐^15^N HSQC spectra at 298 K at 900 MHz spectrometer. First, a reference spectrum of ^15^N‐labeled 150 μM [2Fe‐2S]^2+^ FDX2 (WT and P144L) in 30 mM HEPES, 150 mM NaCl pH 7.5 was acquired. The titration was then performed by increasing the amount of FDXR_ox_ from 0.3 to 1.2 mol eq. To follow the reduction of the [2Fe‐2S] cluster of FDX2 for both WT and P144L proteins by FDXR_ox_/NADPH reducing system, we have prepared four mixtures containing ^15^N‐labeled [2Fe‐2S]^2+^ FDX2 (WT or P144L) and FDXR_ox_ at 1:0.5 and 1:1 ratio with 1 mM NADPH and acquired ^1^H‐^15^N HSQC NMR spectra. The NMR titration data shown are representative of three or more independent experiments.

The observed chemical shift changes observed comparing ^1^H‐^15^N HSQC spectra in Figures 2, 3, and 5 were reported as backbone weighted average chemical shift differences, that is, Δδ_avg_(HN) = (((ΔH)^2^ + (ΔN/5)^2^)/2)^1/2^, where ΔH and ΔN are chemical shift differences for backbone amide ^1^H and ^15^N nuclei, respectively. The estimate of the chemical shift threshold value to define meaningful chemical shift differences was obtained by averaging Δδ_avg_(HN) values plus one standard deviation (1σ), following the standard procedure used in NMR protein–protein interaction studies (Williamson 2013). In the case of the FDX2‐FDXR interaction studies, both chemical shift changes and broadening beyond detection effects, observed in the ^1^H‐^15^N HSQC maps acquired along the NMR titrations, allow us to assign the residues affected by protein–protein interaction.

### 4.4 Isothermal titration calorimetry

Isothermal titration calorimetry (ITC) measurements were carried out at 25°C on the MicroCal iTC200Instrument (GE Healthcare Life Science). Prior to analysis, [2Fe‐2S]^2+^ FDX2 (WT or P144L) and FDXR_ox_ were dialyzed overnight against 50 mM phosphate buffer pH 7.0 in anaerobic conditions. 100 and 90 μL of FDXR_ox_ were placed in the cell of the instrument at a concentration of 20 μM and the solution in the sample cell was stirred at 300 rpm to ensure rapid mixing and heat equilibrium. The titrant, WT (350 μM) [2Fe‐2S]^2+^ FDX2 or P144L (650 μM) [2Fe‐2S]^2+^ FDX2, was inserted into the syringe of the instrument. Twenty additions of 2 μL for WT [2Fe‐2S]^2+^ FDX2 (3.6 μM protein concentration in the cell at the first addition and 61 μM at the last addition), and 28 additions of 0.8 μL for P144L [2Fe‐2S]^2+^ FDX2 each (2.7 μM protein concentration in the cell at the first addition and 69 μM at the last addition), were performed over a period of 10 s with an adequate interval (2–5 min) between injections to allow complete equilibrium. To ensure equilibrium conditions, we wait for the end of the thermal response of the system before making a new addition. To correct for dilution and mixing effects, the control titration, which consisted of the same titration solution, but with buffer in the sample cell, was subtracted from each experiment, accounting for the heat of dilution. The ITC titration syringe and sample cell were cleaned and washed with mild detergent and water after every run. The acquired data were analyzed and fit using the one binding site model incorporated into the Origin 7.0 software supplied with the instrument.

### 4.5 Kinetics of [2Fe‐2S] cluster reduction by FDXR

UV/visible spectra were anaerobically acquired on a Cary 50 Eclipse spectrophotometer in degassed 30 mM HEPES, 150 mM NaCl at pH 7.5. WT or P144L [2Fe‐2S]^2+^ FDX2 (25 μM) were measured in a range of 800–280 nm showing the typical bands of the oxidized [2Fe‐2S]^2+^ cluster. FDXR (3 μM) was anaerobically added to the cuvette and the UV/visible spectrum was acquired as reference. Then, NADPH at a concentration of 300 μM was added into the cuvette and UV/visible spectra were anaerobically acquired each 30 s for 45 min. The peak at 418 nm is diagnostic of the presence of the oxidized [2Fe‐2S]^2+^ FDX2, and it decreases in intensity monitor cluster reduction to [2Fe‐2S]^+^. For the kinetics analysis, the absorbance value at 418 nm was plotted against the time and the curves were fitted with a mono‐exponential decay function.

## AUTHOR CONTRIBUTIONS

**Deborah Grifagni:** Investigation; supervision; visualization; writing – original draft; validation. **Davide Doni:** Investigation; writing – review and editing; data curation; visualization. **Bianca Susini:** Investigation; writing – review and editing; visualization; data curation. **Bruno M. Fonseca:** Investigation; writing – review and editing; visualization. **Ricardo O. Louro:** Investigation; writing – review and editing; visualization. **Paola Costantini:** Conceptualization; writing – review and editing; supervision; funding acquisition; project administration. **Simone Ciofi‐Baffoni:** Conceptualization; funding acquisition; project administration; writing – original draft; writing – review and editing; supervision.

## Supporting information


**Figure S1.** Monitoring [2Fe‐2S]^2+^ cluster reduction of WT and P144L FDX2 followed by paramagnetic NMR and UV/visible spectroscopies.
**Figure S2.** Monitoring thermal unfolding of WT and P144L [2Fe‐2S]^2+^ FDX2.
**Figure S3.** ^15^N relaxation data of WT and P144L [2Fe‐2S]^2+^ FDX2.
**Figure S4.** Analytical size exclusion chromatography of WT and P144L [2Fe‐2S] FDX2.
**Figure S5.** Relaxation rates R_2_ as a function of ν_eff_ measured for both WT and P144L [2Fe‐2S]^2+^ FDX2.
**Figure S6.** ITC measurements of binding of FDXR_ox_ to WT and P144L [2Fe‐2S]^2+^ FDX2.
