# Supplementary material for: Unraveling the molecular determinants of a rare human mitochondrial disorder caused by the P144L mutation of FDX2
Source: Protein Sci. 2024 Oct 28;33(11):e5197. doi: 10.1002/pro.5197 (PMC11515921; doi:10.1002/pro.5197)
Supplement: Supplementary file 1 — Figure S1. Monitoring [2Fe‐2S]2+ cluster reduction of WT and P144L FDX2 followed by paramagnetic NMR and UV/visible spectroscopies. Figure S2. Monitoring thermal unfolding of WT and P144L [2Fe‐2S]2+ FDX2. Figure S3. 15N relaxation data of WT and P144L [2Fe‐2S]2+ FDX2. Figure S4. Analytical size exclusion chromatography of WT and P144L [2Fe‐2S] FDX2. Figure S5. Relaxation rates R2 as a function of νeff measured for both WT and P144L [2Fe‐2S]2+ FDX2. Figure S6. ITC measurements of binding of FDXRox to WT and P144L [2Fe‐2S]2+ FDX2. [file PRO-33-e5197-s001.pdf]

Supplementary Material of

Unraveling the molecular determinants of a rare human mitochondrial disorder caused by the P144L mutation of FDX2

Deborah Grifagni[1,2], Davide Doni[3], Bianca Susini[1,2], Bruno M. Fonseca[4], Ricardo O. Louro[4], Paola Costantini[3,*], Simone Ciofi-Baffoni[1,2,*]

[1]Magnetic Resonance Center CERM, University of Florence, Via Luigi Sacconi 6, 50019, Sesto Fiorentino, Florence, Italy.

[2]Department of Chemistry, University of Florence, Via della Lastruccia 3, 50019, Sesto Fiorentino, Florence, Italy.

[3]Department of Biology, University of Padova, 35121, Padova, Italy.

[4]Instituto de Tecnologia Química e Biológica António Xavier (ITQB-NOVA), Universidade Nova de Lisboa, Av. da República (EAN), 2780-157 Oeiras, Portugal.

Corresponding authors:

*Simone Ciofi-Baffoni, Magnetic Resonance Center CERM and Department of Chemistry, University of Florence, Sesto Fiorentino (Florence) – Italy.

E-mail: ciofi@cerm.unifi.it ; Telephone number: +39 055 4574192

*Paola Costantini, Department of Biology, University of Padova, Padova – Italy.

E-mail: paola.costantini@unipd.it

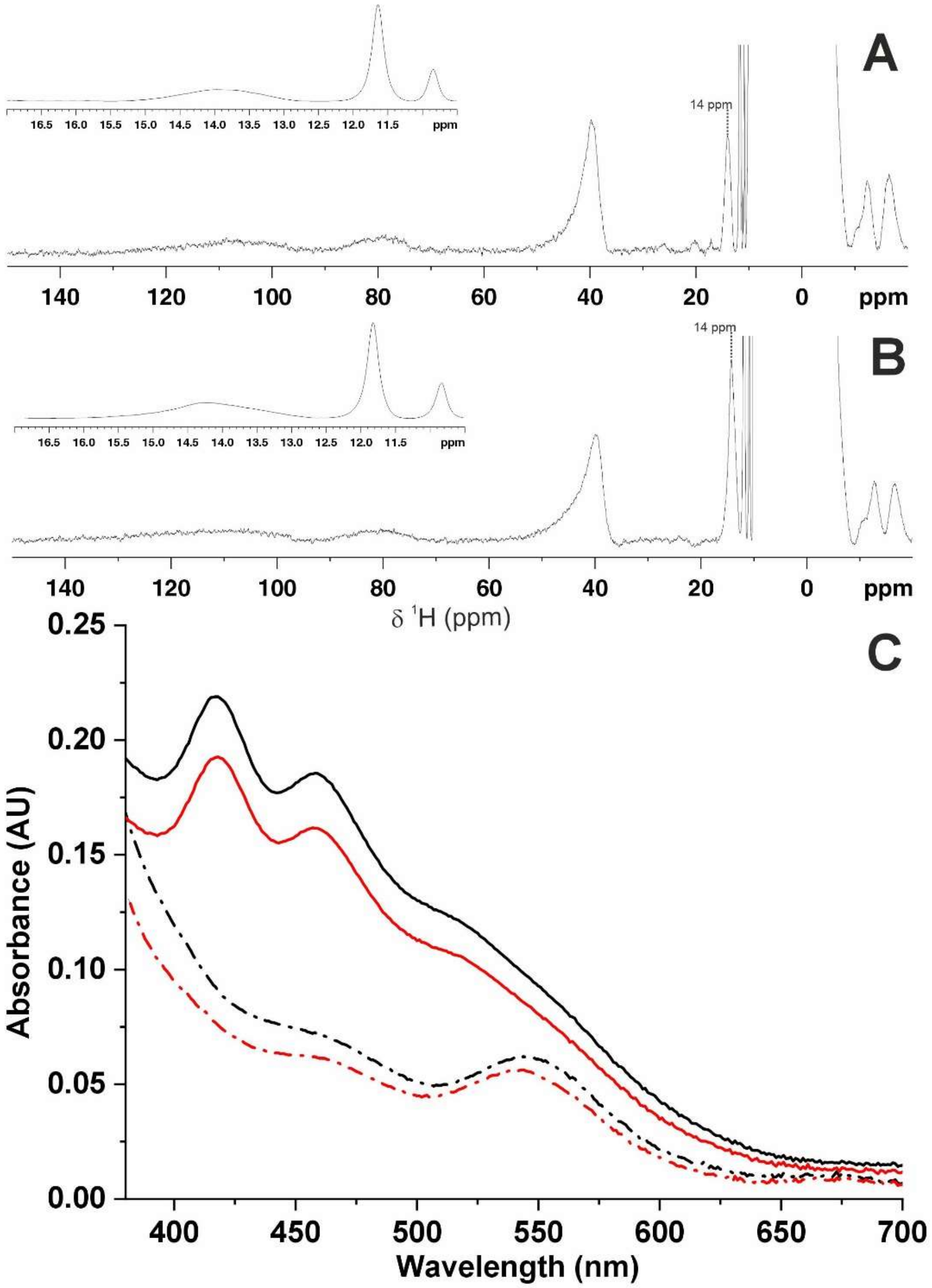

Figure S1. Monitoring $[2Fe\text{-}2S]^{2+}$ cluster reduction of WT and P144L FDX2 by paramagnetic NMR and UV/visible spectroscopies. 1D ^{1}H paramagnetic NMR spectra of P144L (**A**) and WT (**B**) $[2Fe\text{-}2S]^{2+}$ FDX2 reduced by 10 mM dithionite, recorded at 400 MHz and 298 K in 30 mM HEPES buffer, 150 mM NaCl pH 7.5. (**C**) Overlay of UV/visible spectra of P144L (red) and WT (black) $[2Fe\text{-}2S]^{2+}$ FDX2 before (straight line) and after (dashed dotted line) the addition of 10 mM dithionite in 30 mM HEPES buffer, 150 mM NaCl, pH 7.5. Dithionite was removed in glove-box by PD-10 column before acquiring UV/visible spectra.

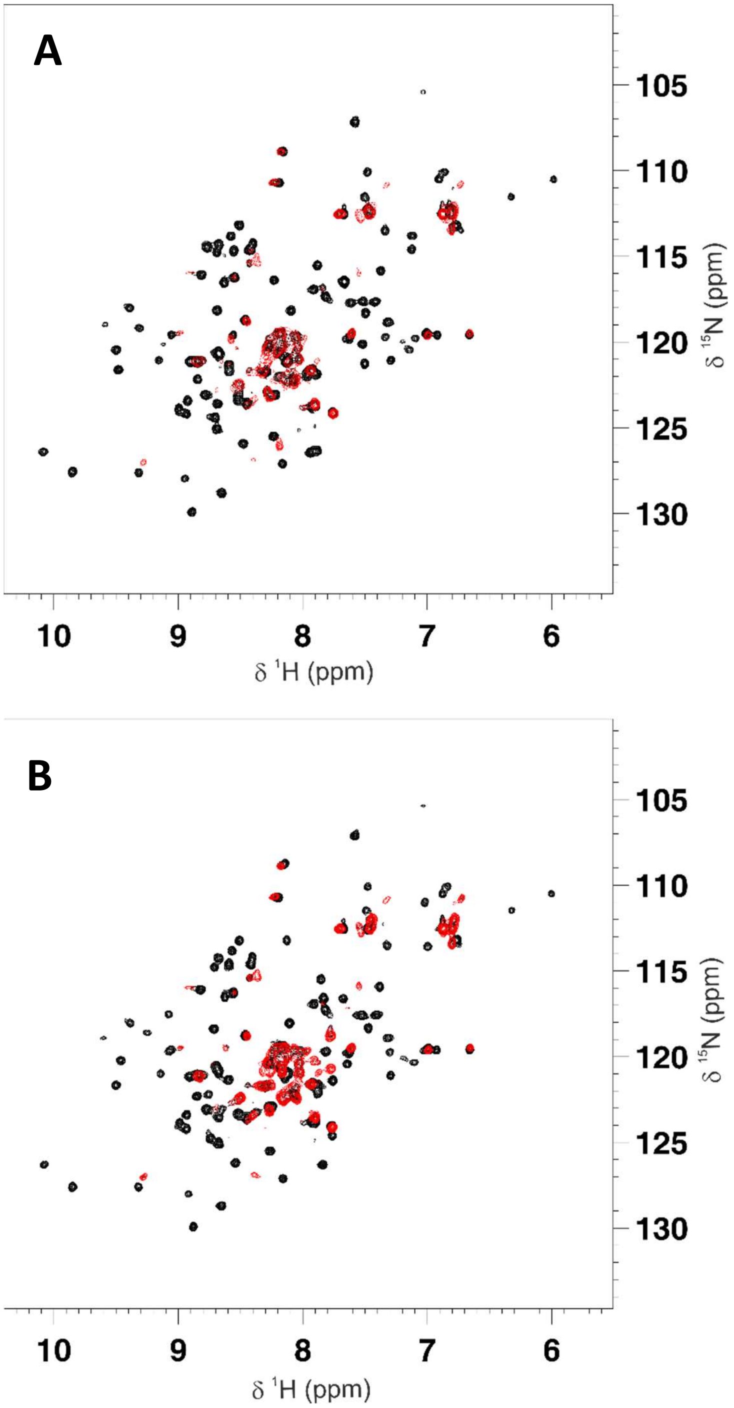

Figure S2. Monitoring thermal unfolding of WT (A) and P144L (B) [2Fe-2S]$^{2+}$ FDX2 by solution NMR. ^{1}H-^{15}N HSCQ NMR spectra were recorded at 298 K, before (black) and after (red) incubating the proteins at 348 K.

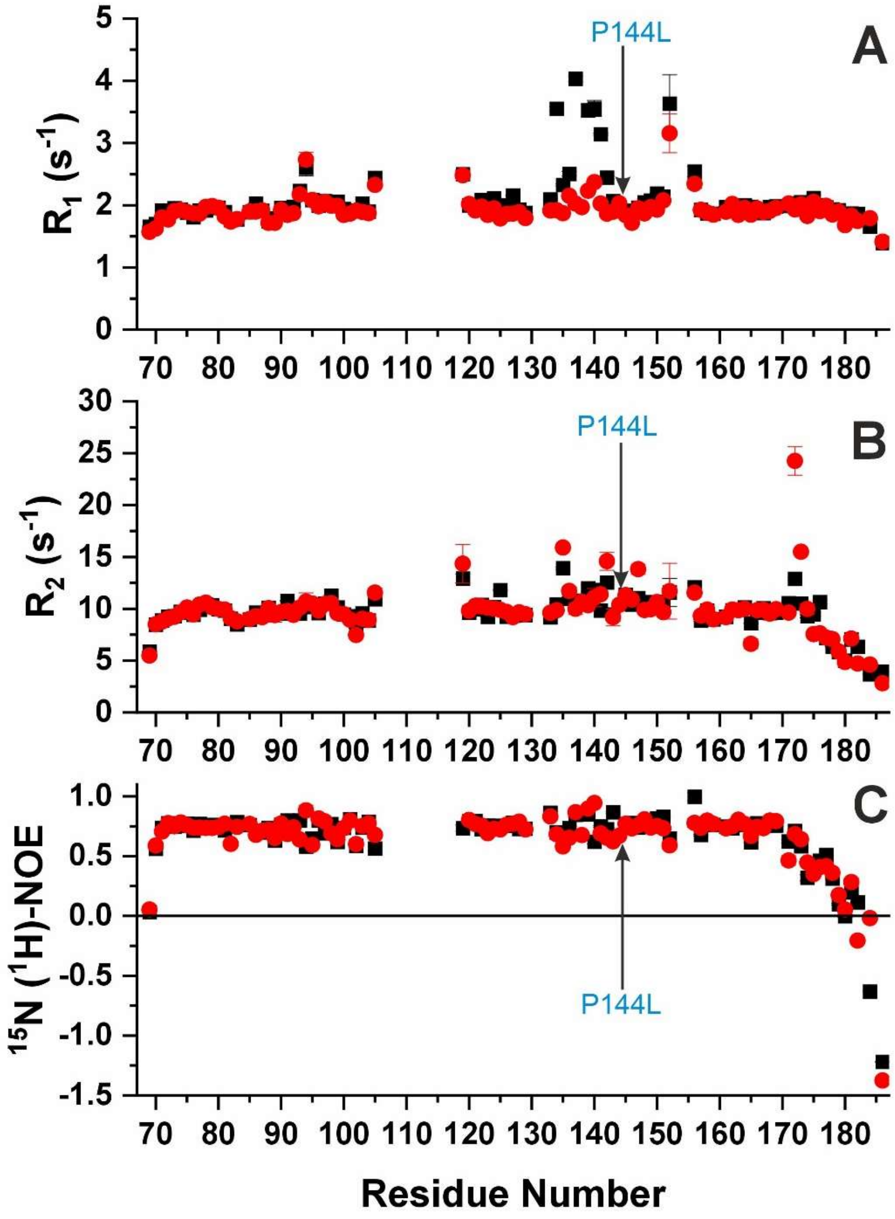

Figure S3. ^{15}N relaxation data of WT and P144L $[2Fe\text{-}2S]^{2+}$ FDX2. ^{15}N longitudinal (R_1) (**A**) and transverse (R_2) (**B**) relaxation rates and $\{^1H\}^{15}N$ heteronuclear NOE (**C**) values of WT (■) and P144L (●) $[2Fe\text{-}2S]^{2+}$ FDX2 determined at 500 MHz and 298 K in 30 mM HEPES buffer, 150 mM NaCl, pH 7.5, and at 800 μM protein concentration. The errors for R_1, R_2 and $\{^1H\}^{15}N$ heteronuclear NOE values were determined directly from the curve fit routine of Bruker Dynamics Center NMR Software.

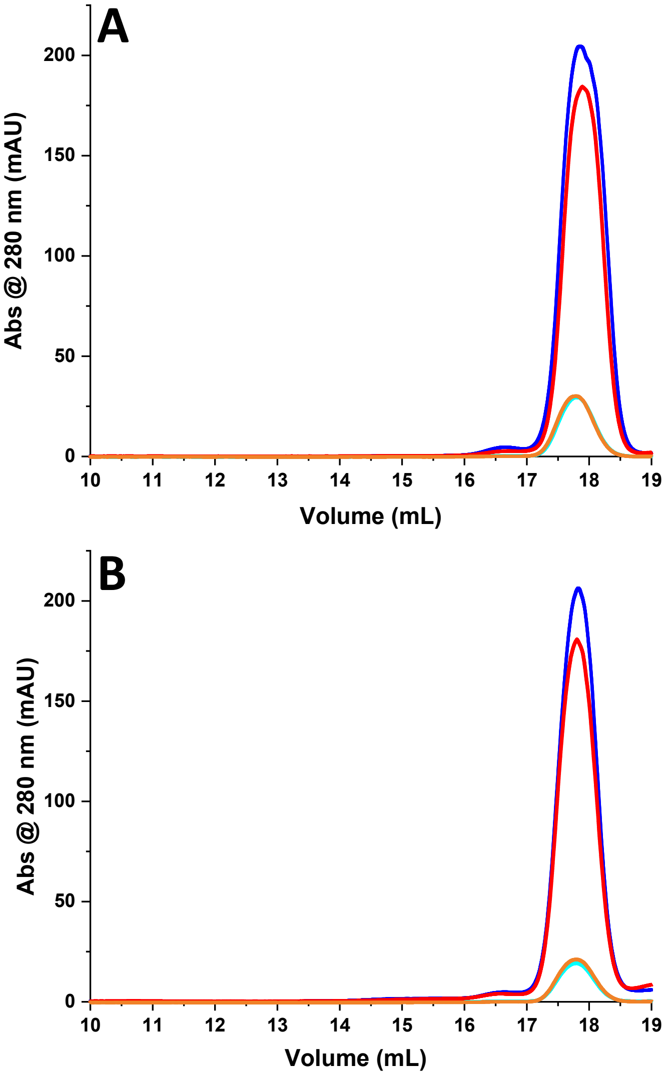

Figure S4. Analytical size exclusion chromatography of WT (A) and P144L (B) [2Fe-2S] FDX2. Elution profiles of WT and P144L oxidized $[2Fe\text{-}2S]^{2+}$ FDX2 at 800 μM (blue) and 80 μM (cyan), and of reduced $[2Fe\text{-}2S]^{+}$ FDX2 at 800 μM (red) and 80 μM (orange).

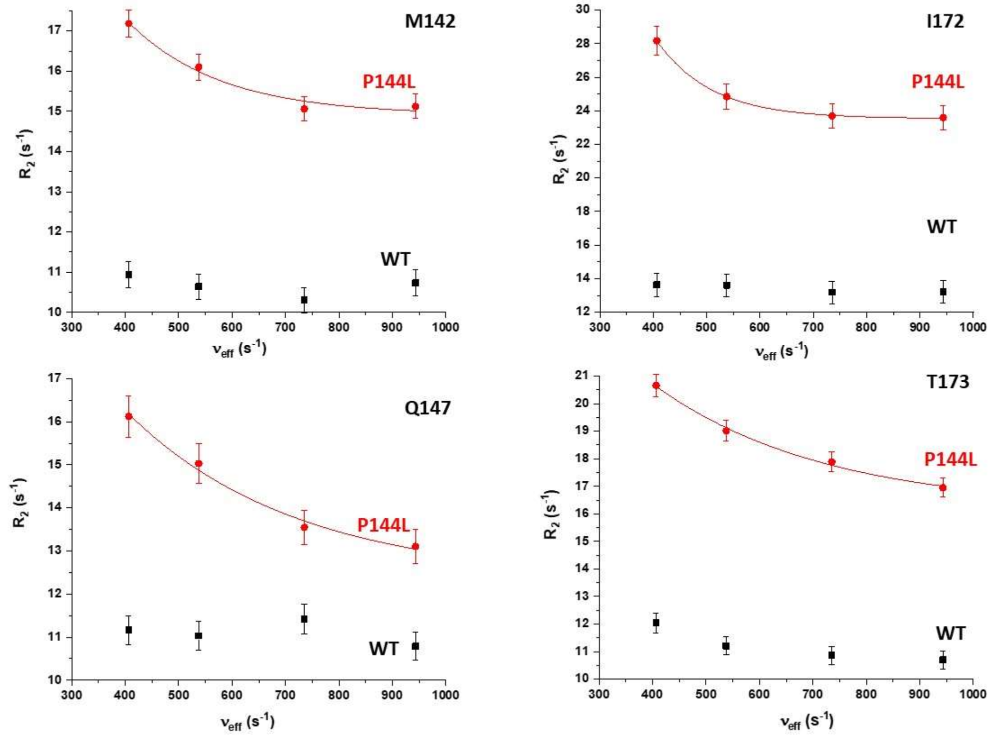

Figure S5. Relaxation rates R_2 as a function of ν_{eff} measured for WT and P144L $[2Fe\text{-}2S]^{2+}$ FDX2. The fittings for residues M142 and Q147, located in the loop containing P144L mutation, and I172 and T174, located in the C-terminal tail in contact with P144, are reported for P144L $[2Fe\text{-}2S]^{2+}$ FDX2. The correlation times of the exchange process, τ_{ex}, obtained by these fittings, are respectively for the four residues: 173, 315, 103 and 318 μs. No significant decay has been detected in the WT protein.

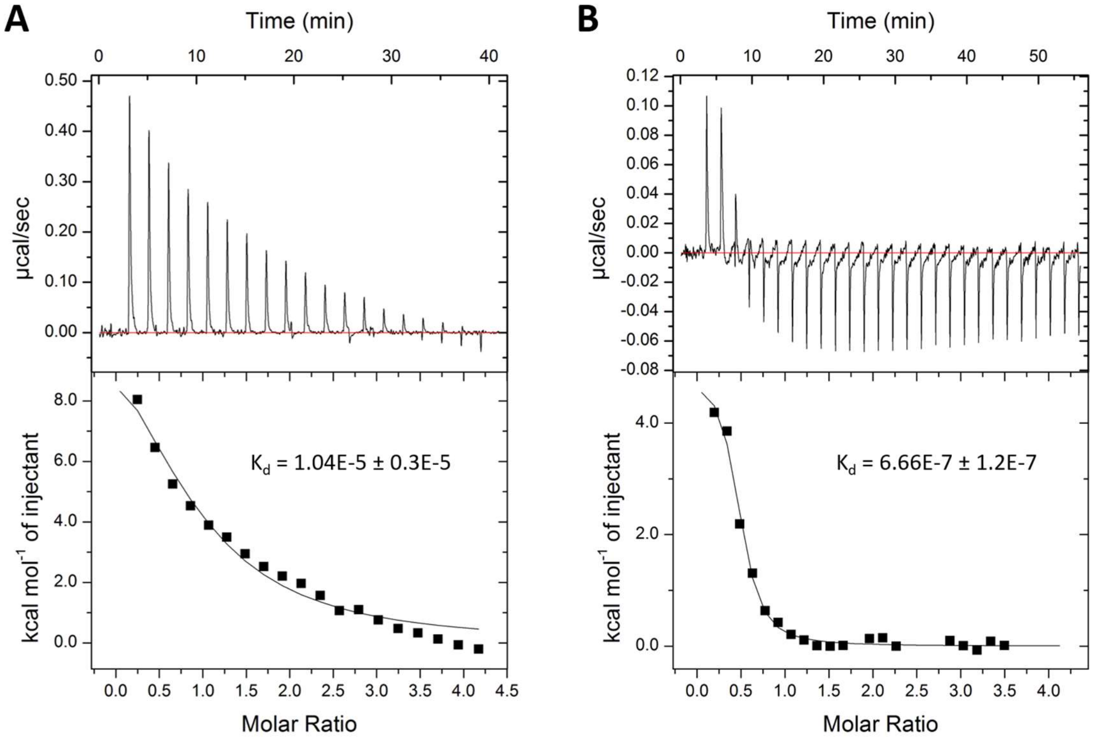

Figure S6. ITC measurements of binding of FDXR$_{ox}$ to WT and P144L $[2Fe\text{-}2S]^{2+}$ FDX2. (**A**) ITC assay between FDXR$_{ox}$ and WT $[2Fe\text{-}2S]^{2+}$ FDX2. (**B**) ITC assay between FDXR$_{ox}$ and P144L $[2Fe\text{-}2S]^{2+}$ FDX2. The baseline-leveled raw ITC data corresponds to the upper panel, and the fitting to the resultant titration curve using a single binding site model corresponds to the lower panel. The obtained dissociation constant (K_d) is shown in the lower panel of each ITC assay. The experiments were performed at 25°C in 50 mM phosphate buffer, pH 7.0.
